# TRAIL‐PEG‐Apt‐PLGA nanosystem as an aptamer‐targeted drug delivery system potential for triple‐negative breast cancer therapy using *in vivo* mouse model

**DOI:** 10.1002/1878-0261.70202

**Published:** 2026-02-08

**Authors:** Gulen Melike Demirbolat, Aslihan Kucuk, Omer Erdogan, Samed Ozer, Bensu Kozan, Tugba Cuceli, Erkan Gumus, Evrim Cevik, Ozge Cevik

**Affiliations:** ^1^ Department of Pharmaceutical Technology Faculty of Pharmacy, Acibadem Mehmet Ali Aydinlar University Istanbul Turkey; ^2^ Department of Molecular Biotechnology Graduate School of Health Sciences, Aydin Adnan Menderes University Turkey; ^3^ Department of Medicinal Biochemistry School of Medicine, Gaziantep Islam Science and Technology University Turkey; ^4^ Animal Application and Research Center Acibadem Mehmet Ali Aydinlar University Istanbul Turkey; ^5^ Department of Histology and Embryology School of Medicine, Aydin Adnan Menderes University Turkey; ^6^ Department of Machine and Metal Technologies Kocarli Vocational School, Aydin Adnan Menderes University Turkey; ^7^ Department of Medicinal Biochemistry School of Medicine, Aydin Adnan Menderes University Turkey

**Keywords:** aptamers, breast cancer, PLGA copolymers, targeted therapy, TRAIL

## Abstract

Targeted drug therapy is very important for the treatment of triple‐negative breast cancer (TNBC), and the development of carrier systems to deliver apoptosis‐inducing proteins such as TRAIL to cells is important in cancer therapy. In this study, a nanosystem formulation (TRAIL‐PEG‐Apt‐PLGA) encapsulating TNBC‐targeted aptamer‐bound‐TRAIL protein was performed and the efficacy of this system was evaluated in a mouse tumor model. The characterization of TRAIL‐PEG‐Apt‐PLGA was confirmed by FTIR, NTA and SEM microscopy. The efficacy of TRAIL‐PEG‐Apt‐PLGA was evaluated by *in vitro* release assays and interactions with TNBC cells (MDA‐MB‐231) and healthy breast cells (MCF‐10A). TRAIL‐PEG‐Apt‐PLGA was administered intravenously to NOD/SCID gamma mouse breast tumors and evaluated *in vivo*. Pharmacokinetics, bioavailability testing, histological staining (DR4/DR5, TUNEL, HE staining) and molecular alterations with PCR array were evaluated in tumor tissues. TRAIL‐PEG‐Apt‐PLGA induced apoptosis in both *in vivo* and *in vitro* studies. It was found that it regulated cellular responses along with apoptotic mechanisms in cells without developing resistance in suppressing tumor growth by making changes on Atf2, Casp8, Bcl2 and Irf5 genes and proteins. As a result, the biotechnological drug potential of TRAIL was discovered in an aptamer‐bound nanosystem for the treatment of triple‐negative breast cancer and innovative applications for clinical use.

## Graphical abstract

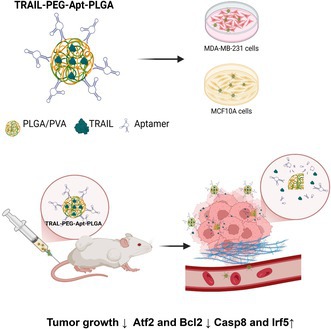

**Abbreviations**
Atf2: Activating transcription factor 2
Bcl‐2: Bcl‐2‐associated X protein
Cas8: Caspase‐8
DR4 and DR5: Death receptor 4 and 5
FDA: United States Food and Drug Administration
IRF5: Interferon regulatory factor 5
PLGA: Poly‐lactic‐co‐glycolic acid
PTX: Paclitaxel
PVA: Polyvinyl alcohol
TRAIL: Tumor necrosis factor‐related apoptosis‐inducing ligand

## 1 Introduction

Tumor necrosis factor‐related apoptosis‐inducing ligand (TRAIL) is a highly active protein that has been shown to bind to death receptors from the tumor necrosis factor (TNF) family. It has been reported that TRAIL triggers apoptosis when it binds to its receptor within the cell. While its impact on healthy cells is less pronounced, its proapoptotic effect on cancer cells renders TRAIL a significant target for cancer treatment [1]. TRAIL exerts its effects by binding to receptors located on the cell surface. Additionally, TRAIL has been found to be present in the plasma in a free state [2]. TRAIL‐1 (DR4) and TRAIL‐2 (DR5) receptors are reported to be highly expressed on cancer cells. Therefore, DR4 and DR5 are targeted in drug development studies for cancer treatment [3]. When the TRAIL ligand binds to DR5 or DR4 receptors, it activates the apoptotic pathway [3]. It is known that TRAIL injected intravenously is effective after 5 h and inhibits tumor growth to a large extent according to pharmacokinetic studies. Studies have shown that it has an antitumor effect when injected locally with chemotherapeutic agents or when targeted to tumor tissue [4, 5, 6]. However, formulations are needed for cancers where TRAIL is difficult to target, such as triple‐negative breast cancer (TNBC, HER, PR and ER). Since targeting surface receptors is not possible in cancers such as TNBC, more specific, innovative targeting agents are needed.

Aptamers are composed of short single‐stranded DNA or RNA strands that fold in a specific manner, with a length ranging from 20 to 100 bases [7]. The stability of their structures is attributed to weak interactions, including hydrophobic interactions, Van der Waals forces, and electrostatic interactions [8]. In their interactions with the target molecule, these weak interactions and selection are provided as in the key/lock fit [9]. Due to their low molecular weight and structure, aptamers have been demonstrated to be highly effective in reaching target molecules upon entering the cell. In comparison to antibodies, aptamers exhibit several advantages, including low production costs, short production times, thermal stability, minimal immune response, and lyophilizability [10, 11, 12]. In recent years, the use of aptamers as ligands in therapeutic applications and recognition of cancer structures in cancer treatment has been a remarkable development [13]. Poly(lactic‐co‐glycolic acid) (PLGA)‐containing systems (such as PLGA nanospheres, PLGA microspheres), which are one of the nano‐sized systems, provide extended release. Extended release can be observed with lower dosing frequency and increased efficacy without toxicity [14, 15]. Biodegradable polymers such as PLGA are widely used due to their controlled degradation rate, biocompatibility, and approval by the United States Food and Drug Administration (FDA). PLGA‐containing nanoparticles are generally prepared by two methods: nanoprecipitation and solvent evaporation. In nanoprecipitation, the polymer is dissolved in the organic solvent and then added to the aqueous phase, and nanoparticle structures are formed after displacement. In this method, the encapsulation of hydrophobic drugs is generally higher. In the water/oil/water (w/o/w) double emulsion/solvent evaporation method, the polymer and drug are dissolved in the organic solvent in the presence of the aqueous phase, and the solvent is evaporated by a high‐pressure homogenizer after emulsification. In this method, the encapsulation capacity is higher, especially for hydrophilic drugs [16]. Therefore, in this study, PLGA nanoparticles were developed as a drug delivery system. Since the aptamer is selective for cells and TRAIL will show antitumor activity, PLGA nanoparticles were determined to be the necessary carrier system for the combined use of TRAIL and aptamer in this study.

## 2 Material and methods

### 2.1 Production and purification of recombinant TRAIL

The protein region between Thr(95)‐Gly(281) amino acids is produced for TRAIL. For this region in soluble form, pQE‐hTR was a gift from Wafik El‐Deiry (Addgene plasmid # 21811; RRID:Addgene_21811). The pQE‐hTR plasmid was placed in BL21 bacteria and used as competent bacteria for protein production. Subsequently, 2 μL of plasmid DNA and 100 μL of BL21 bacterial suspension in TE buffer were maintained on ice for a duration of 10 min. Thereafter, 0.2 cm special electroporation cuvettes were positioned, and a voltage of 1.72 kV at 25 μF was applied with an electroporator (Biorad Gene Pulser, Hercules, CA, USA). Following electroporation, a liquid SOC medium (tryptone 20 g/L, yeast extract 5 g/L, NaCl 0.5 g/L, 250 mm KCl 1 mL/L) was added to the suspension and incubated at 200 rpm for 1 h at 37 °C. Following this, the prepared plasmids, which exhibited ampicillin selectivity, were plated on solid LB‐agar medium. These plates were then incubated overnight at 37 °C. Positive colonies were selected and cultured in LB liquid medium containing amp (tryptone 10 g/L, yeast extract 5 g/L, NaCl 10 g/L). The propagation of these cells was achieved through shaking incubation at 37 °C for an overnight period. To induce the expression of the desired protein, a concentration of 0.5 mm IPTG was utilized, with the incubation temperature maintained at 37 °C. Following the induction step, the induced E. coli cells were subjected to centrifugation at 800 ***g*** for a duration of 15 min at a temperature of 4 °C. The ruptured bacterial pellets were then subjected to lysis buffer (50 mm sodium phosphate, pH 8.0, 300 mm NaCl, 10 mm imidazole, 10 mm β‐mercaptoethanol, 5 mm dithiothreitol, and protease inhibitors) application via ultrasonic waves at 4 °C. The purification of TRAIL protein (Fig. S1) was achieved through the use of a Ni‐NTA IMAC 16 mm × 25 mm column system (Biorad, NGC 10 Chromatography Systems).

### 2.2 Cell culture conditions

The breast cancer cell line MDA‐MB‐231 (RRID: CVCL_0062) and the control breast cell line MCF‐10A (RRID: CVCL_0598) were procured from the American Type Culture Collection (ATCC). The culture of MDA‐MB‐231 cells was performed in DMEM medium containing 10% fetal bovine serum, 1% penicillin and streptomycin, and L‐glutamine. For MCF‐10A, DMEM medium containing 5% horse serum, 10 μg·mL^−1^ insulin, 20 ng·mL^−1^ EGF, 0.5 μg·mL^−1^ hydrocortisone, 1% penicillin/streptomycin, and L‐glutamine was used. The cells were then cultivated at 37 °C within an incubator containing 5% carbon dioxide. Subsequent to achieving a specific degree of cell confluence, the cells were passaged. All commercial cell lines were validated by short‐tandem repeat (STR) sequencing (Gen Era Diagnostic, Turkey) in the past 3 years. Cells were regularly screened for mycoplasma contamination (Promocell, Heidelberg, Germany) according to the manufacturer's protocol. All experiments were performed with mycoplasma‐free cells.

### 2.3 Aptamer binding experiments

The LXL‐1‐A (GAATTCAGTCGGACAGCGAAGTAGTTTTCCTTCTAACCTAAG AACCCGCGGCAGTTTAATGTAGATGGACGAA) aptamer, which has been demonstrated to be effective for targeting breast cancer [17], was selected. MDA‐MB‐231 and MCF‐10A cells, grown in 25 cm^2^ flasks to 80% confluency, were enzymatically detached using trypsin–EDTA, followed by a wash with phosphate‐buffered saline (PBS). The cell count was performed using a cell counter, and the cells were subsequently placed in eppendorf tube at a concentration of 2 × 10^5^ cells·mL^−1^. The cells were then suspended in binding buffer (100 mm NaCl, 5 mm MgCl_2_, 50 mm Tris/HCl, pH 7.2) and incubated with different concentrations of aptamer (100 pmol and 500 pmol) on ice for a duration of 1 h. Subsequently, the cells were subjected to a centrifugal process at 2500 ***g*** for a duration of 5 min, resulting in the removal of unbound aptamers from the medium. Subsequent washing procedures were executed with phosphate‐buffered saline (PBS), and this washing procedure was repeated twice more with PBS for 5 min at 2500 ***g***. Subsequently, FITC‐conjugated aptamers were identified through the implementation of FSC and SSC sizing in a flow cytometer (Agilent Novocyte, Santa Clara, CA, USA). A total of 100 000 cells were enumerated, and the presence of aptamers on the cell surface was determined within the FITC panel.

Fluorescence reader (Thermo Scientific Fluoroskan Ascent Reader, Vantaa, Finland) was used to calculate the binding levels of aptamers to MDA‐MB‐231 and MCF‐10A cells at different concentrations and to determine the *K*_d_ value. 1 × 10^4^ cells were counted and placed in Eppendorf tubes and incubated with LX1A‐FITC‐labeled aptamer at a concentration of 0–200 nm for 30 min at room temperature in the dark. After incubation, the cells were centrifuged at 2500 ***g*** for 5 min and washed once with PBS to remove unbound aptamer. Then 200 μL of the cell suspension was added to the black plate and read by selecting 485/520 filters as *E*_x_/*E*_m_ and fluorescence intensity values were recorded.

### 2.4 PEG‐aptamer synthesis

Using amine modified aptamer and carboxy modified PEG, aptamer and PEG were linked to each other with EDC and NHS crosslinking agents. In attaching LX1A aptamer to PEG structure, the aptamer was attached via amine groups. PEG‐COOH was prepared in DNase and RNase‐free water at a concentration of 10 μg·μL^−1^. 400 mmol/L 1‐ethyl‐3‐(3‐dimethylaminopropyl) carbodiimide hydrochloride (EDC) reagent and 100 mmol/L N‐Hydroxysulfosuccinimide (NHS) were added and incubated at room temperature for 30 min [7]. Then, it was treated with modified LX1A‐NH2 aptamer and incubated at room temperature for 3 h. After conjugation, unbound molecules were separated by centrifugation at 15 000 rpm for 30 min. Washing was done with DNase‐RNase‐free water and centrifuged again at 15 000 rpm for 30 min and suspended. FTIR analysis was performed by evaporating the aqueous part in the lyophilizer.

### 2.5 Preparation of TRAIL‐loaded PLGA nanoparticles

The double emulsion solvent extraction method was employed for the preparation of nanoparticles, which is based on w/o/w type emulsions [18, 19, 20]. PLGA was dissolved in acetone, and TRAIL was dissolved in a sodium acetate solution with a pH of 5.0. The TRAIL solution was then introduced into the PVA aqueous solution, and the mixture was placed on a magnetic stirrer at 500 rpm. Concurrently, the PLGA solution was introduced dropwise, at a height of 5 cm, to the PVA aqueous solution containing TRAIL, employing a 30G insulin syringe. The prepared formulations were then placed on ice packs and homogenized with a 30% amp ultrasonic probe for 1 min (2 s on, 1 s off). Subsequently, the formulations were left under stirring at 500 rpm for 24 h at room temperature in the dark without applying heat in order for the acetone to evaporate. Following the evaporation of the acetone, the samples were returned to ice packs and stirred at 70% amplitude with a 1‐min ultrasonic probe homogenization cycle (2 s on, 1 s off). Ultracentrifugation at 30 000 rpm at 4 °C for 30 min was then employed to sediment the samples. The resulting supernatants were then separated for quantification. The sediment was dispersed with 1 mL of distilled water and stored in a refrigerated environment for subsequent use. (Tables S1 and S2).

### 2.6 Characterization of TRAIL‐loaded PLGA nanoparticles

In order to evaluate the particle size and distribution on the samples, particle sizes and polydispersity indices (PDI) were measured using the particle size analyzer Litesizer 500 (Anton Paar, Australia) based on the dynamic light scattering method. The measurements were conducted at a temperature of 25.0 ± 0.1 °C using 660‐nm laser light at a 90° angle and six parallel lines, following the dilution of the samples by a factor of 200 with distilled water. At the same time, the morphological examination of the nanoparticle structure was performed using a scanning electron microscope (SEM).

### 2.7 Investigation of solubility and partition coefficient of TRAIL protein

TRAIL‐loaded and unloaded PLGA in pH 7.4 phosphate‐buffered saline (PBS) buffer was measured with a pH meter at 25 °C and 37 °C and over time [21]. The active substance was added in portions to 5 milliliters of buffer solution, and the results were subsequently determined using a UV spectrophotometer to ascertain the solubility of the active substance. The operating conditions were established as sink conditions, and release studies were conducted in accordance with USP29 guidelines.

A specific quantity of TRAIL protein was incorporated into an octanol: water mixture, and following an overnight wait, the phase and ratio in which it was present were ascertained [22]. This approach enabled the assessment of TRAIL protein permeability from the internal phase (aqueous portion) to the PLGA structure.

### 2.8 Loading capacity measurement, dissolution studies and stability tests

To assess the loading capacity, TRAIL‐loaded PEG‐Aptamer PLGA nanoparticles were dissolved in a NaOH solution and maintained at 37 °C for a period of 4 h [19]. Subsequently, the samples were subjected to centrifugation, and the resulting supernatant was quantitatively analyzed to determine the concentration of TRAIL protein. To assess the dissolution profile of the nanoparticles, TRAIL‐loaded nanoparticles, empty nanoparticles, and TRAIL‐PEG‐aptamer nanoparticles were meticulously dispensed into a series of Eppendorf tubes containing pH 7.4 PBS buffer. Tween‐20 was incorporated into the medium, and the Eppendorf tubes were subjected to agitation at 37 °C for varying durations ranging from 0 to 72 h. Subsequent to the dissolution study, the nanoparticles within the collected samples were subjected to centrifugation, and the resulting supernatant was quantitatively analyzed for TRAIL protein. Quantifications were determined using the Human TRAIL ELISA kit (ELK1320), a commercial kit. Three replicate analyses were performed on each sample, and TRAIL was calculated as ng·mL^−1^. The relevant stability tests of the biotechnological drug candidate were planned in accordance with the ICH Q1A guideline. Given that the TRAIL‐PEG‐Apt‐PLGA nanoparticle formulation loaded with the active substance was to be maintained in both water and phosphate buffer during the development and quality control stages, the stability of the formulation at 25 °C, 30 °C, and 40 °C ± 2 in these environments, as well as its stability at 37 °C during the study period in BSA, was evaluated. This evaluation was conducted with the aim of assessing the stability of the formulation within the blood circulation system. The evaluation of stability was conducted through two methodologies: particle size measurement and TRAIL quantity determination.

### 2.9 TRAIL‐PEG‐apt‐ PLGA nanoparticles cell surface binding studies

MDA‐MB‐231 cells were seeded in 6‐well plates at a density of 1 × 10^6^ cells per well. Plain DMEM (no FBS or antibiotics) was added to the cells. TRAIL‐PEG‐Apt‐PLGA (10 μg·mL^−1^ and 40 μg·mL^−1^) from particles containing FITC‐conjugated aptamer was added on top of the cells. IgG‐FITC (5 μL, sc‐2855) was utilized as a control to detect non‐specific binding. Following the preparation of the samples, they were subjected to incubation for 30 min and 120 min. Thereafter, they were subjected to centrifugation at 500 ***g*** for 10 min to remove unbound cells. The resultant pellet was discarded. The cells that remained at the bottom of the tube were then washed once with phosphate‐buffered saline (PBS), after which they were suspended in fresh PBS. The cells were subsequently measured in the FITC panel of the flow cytometer (Agilent Novocyte). A total of 10 000 cells were counted, and the gate was determined for the cells. The binding levels were then calculated.

### 2.10 Cytotoxicity studies of the TRAIL‐PEG‐apt‐ PLGA nanoparticles

MDA‐MB‐231 and L929 cells were seeded into 96‐well plates at a density of 1.5 × 10^4^ cells per well. The plates were then maintained overnight at 5% CO_2_ and 37 °C to allow the cells to adhere and reach sufficient numbers. Subsequently, the formulations were added at concentrations of 0.1, 1, 10, 100, and 1000 μg·mL^−1^, with dimethyl sulfoxide (DMSO) serving as the control group. The cells were then incubated for a period of 24 h. Thereafter, MTT solution was added, and the incubation was continued for an additional 4 h. Subsequently, solubilization buffer was added to the medium to dissolve the tetrazolium salts. After a 30‐min waiting period, readings were obtained using an ELISA reader (Epoch, BioTek Instruments, Winooski, VT, USA) at a wavelength of 570 nm. The calculation of IC50 values and the evaluation of cell morphologies were subsequently conducted.

### 2.11 TRAIL‐PEG‐apt‐ PLGA nanoparticles cell colony formation studies

MDA‐MB‐231 cells were seeded in 6‐well plates at a density of 1000 cells per well. TRAIL (10 ng·mL^−1^), TRAIL‐PEG‐Apt‐PLGA (5, 10, 20 μg·mL^−1^), and paclitaxel (10 nm) were added to the cells and incubated for a period of 14 days. The medium was changed every 3 days, and fresh drug molecules were added. Subsequently, the medium was discarded and fixation was performed with acetic acid methanol (3 : 1) to observe the colonies formed. The fixed cells were then stained with 0.5% crystal violet dye for 15 min, after which the plate was washed under water. Subsequently, images of the cell plates were captured, and the colonies were examined under an inverted microscope. The colony counts were performed using the ImageJ software and calculated as a percentage. Each experiment was independently repeated three times.

### 2.12 TRAIL‐PEG‐apt‐ PLGA nanoparticles apoptotic studies

MDA‐MB‐231 cells were seeded in 6‐well plates at a density of 1 × 10^6^ cells per well. TRAIL (10 ng·mL^−1^) and TRAIL‐PEG‐Apt‐PLGA (10 μg·mL^−1^), the positive control cancer drug paclitaxel (10 nm) was also included in the assay. The cells were then incubated for 24 h. Following the incubation period, the cell medium was discarded and the cells were washed twice with PBS. The cells were then detached using trypsin EDTA, and the cell pellets were obtained by centrifugation at 500 ***g*** for 10 min. The cells were then prepared using the Annexin‐V‐FITC kit (BD Pharmigen, 556570) according to the manufacturer's procedure and suspended with an annexin binding buffer.100 μL of cell suspension was taken and annexin‐V (5 μL) and PI (5 μL) were added. The mixture was then incubated at room temperature in the dark for 30 min. Subsequently, 20 000 cells were enumerated using flow cytometry (Novocyte, Agilent) to establish a gate, and apoptotic and living cells were subsequently analyzed.

### 2.13 Pharmacokinetic studies of TRAIL‐PEG‐apt‐PLGA nanoparticles

For the pharmacokinetic studies, a single dose of the formulation was administered intravenously to female Balb‐c mice weighing 30 g. Blood was collected by cutting their tails at specific times. TRAIL (100 ng·kg^−1^), TRAIL‐PEG‐Apt‐PLGA (100 μg·kg^−1^) was administered, and a PBS solution was utilized as a control. The applications were completed in the mice. Subsequent to drug administration, blood was extracted from the tail vein into tubes containing EDTA at 30 min, 60 min, 2 h, 4 h, 8 h, and 24 h, and plasma was separated by centrifuging the blood samples at 2000 rpm for 5 min. TRAIL levels in the plasma were subsequently quantified using an enzyme‐linked immunosorbent assay (ELISA). Pharmacokinetic parameters, including Cmax (maximum blood concentration), tmax (time to reach maximum blood concentration), AUC (area under the curve), and t1/2 (half‐life), were calculated.

### 2.14 *In vivo* breast cancer mouse model and TRAIL‐PEG‐apt‐PLGA nanoparticles efficacy tests

The Acibadem University Experimental Animals Local Ethics Committee for *in vivo* studies (ACU‐HADYEK 2023/68) provided its approval for the aforementioned studies. All animal experiments were performed in accordance with protocols approved by the Acibadem Mehmet Ali Aydinlar University Laboratory Animal Application and Research Center (ACUDEHAM) following Good Laboratory Practices for Animal Research. NOD/SCID mice were housed in a specific pathogen‐free (SPF) facility in individually ventilated cages at 20–24 °C, 40–60% humidity and a 12 : 12 h light–dark cycle. Sterile food and water were provided *ad libitum*. Animals were handled under aseptic conditions and monitored daily for health status and tumor burden.

NOD/SCID gamma mice (NSG) were utilized in the experimental studies for the *in vivo* tumor model. The experimental design involved the formation of three groups, with eight animals (*n* = 8) allocated to each group. All applications were carried out in a sterile environment in a biosafety type 2 cabinet. MDA‐MB‐231‐Luc breast cancer cells were mixed with matrigel (1 : 1) subcutaneously on the right hind leg of NSG mice (5–7 weeks old female, weighing 25–40 g) and injected into the mice with 0.1 mL of 5 × 10^6^ cells to form tumors. After 25 days, the growth of the tumors was detected in the mice, and the formulations were injected. The mice were weighed, and the tumors were measured with a caliper. The Breast Cancer Group conducted the following experiment: MDA‐MB‐231‐Luc cells, which are a model of breast cancer, were injected into the mammary fat pad of the mouse once with a solution of DMEM and matrigel (0.1 mL). After the formation of a palpable tumor, the mice were treated with PBS (0.1 mL, intravenously) three times and observed for a period of 15 days. The TRAIL group underwent the following protocol: MDA‐MB‐231‐Luc cells for breast cancer were injected once with DMEM matrigel (0.1 mL) and after tumor formation, TRAIL (100 ng·kg^−1^, intravenously) was administered three times and followed for 15 days. The TRAIL‐PEG‐Apt‐PLGA group received the following treatment: MDA‐MB‐231‐Luc cells for breast cancer were injected with DMEM matrigel once (0.1 mL) and after tumor formation, TRAIL‐PEG‐Apt‐PLGA (100 μg·kg^−1^, iv) was administered three times and followed for 15 days. The tumor growth was subsequently analyzed at the conclusion of the experiment using the IVIS imaging system. The percentage of tumor shrinkage (volume = L*W*H)/2 was calculated. At the end of the experiments, blood samples were taken from the mice and tumor tissues were removed.

### 2.15 Histological analyses on mouse tumor tissues

After the tumor tissues were fixed in 10% formaldehyde, they were tracked using an automatic tissue tracker (Leica TP1020). After deparaffinization through a series of alcohols, hematoxylin and eosin staining was performed. To identify DNA breaks *in situ*, the presence of apoptosis in the cells was determined using the TUNEL method and specific staining was performed using DAB (*In Situ* Cell Death Detection Kit, Roche Diagnostics, Mannheim, Germany; Cat. No. 11684817910). After tumor tissue tracking [23], immunohistochemical (IHC) staining for DR4 and DR5 was performed on the collected sections. Incubation was performed overnight with primary antibodies anti‐DR4 (1 : 100) and anti‐DR5 (1 : 100) in substrate chromogen 3,3′‐diaminobenzidine (DAB, Thermo TA‐125‐QHDX) solution. Mounting medium (Abcam, Cambridge, UK, Cat. No. ab64230) was added to the specimen, covered with a coverslip, and images were captured under a microscope (Zeiss Axiovert1).

### 2.16 RT2 profiler PCR array profiling studies

In this study, RNA was isolated from three different tumor tissues from each group using a commercially available kit (Qiagen, Hilden, Germany Cat. No. 74106, Rneasy Mini Kit) and cDNA synthesis was performed using a kit (Qiagen 205314, QuantiTect Rev Transcription Kit). For gene expression profiling using RT^2^ Profiler PCR Arrays, Cell Cancer Drug Targets PCR Array (PAMM‐507Z, Qiagen) and Oncogenes and Tumor Suppressor Genes PCR Array (PAMM‐502ZA, Qiagen) were commercially purchased in the obtained cDNAs and analyzed using sybr green (Qiagen 330500 RT^2^ SYBR Green qPCR Mastermix) on a qPCR instrument (ABI, StepOnePlus). Each array was studied in three replicates. For genes where differences were observed in gene expression profiling, quantification was performed by qPCR with specific primers using SYBR Green dye. In the present study, the authors utilized mammary tissue samples obtained from mice to serve as a control group in the profiling analysis. Analyses were performed in the tumor group, the TRAIL treatment group, and the TRAIL‐PEG‐Apt‐PLGA treatment group according to the control group and by comparing them with each other. Five genes were selected as housekeeping genes (Actb, B2m, Gapdh, Gusb, Hsp90ab1) and utilized for the analysis. Genes exhibiting 2‐fold and greater changes were selected for further analysis.

## 3 Results

### 3.1 Aptamer binding experiment results

**Fig. 1:**
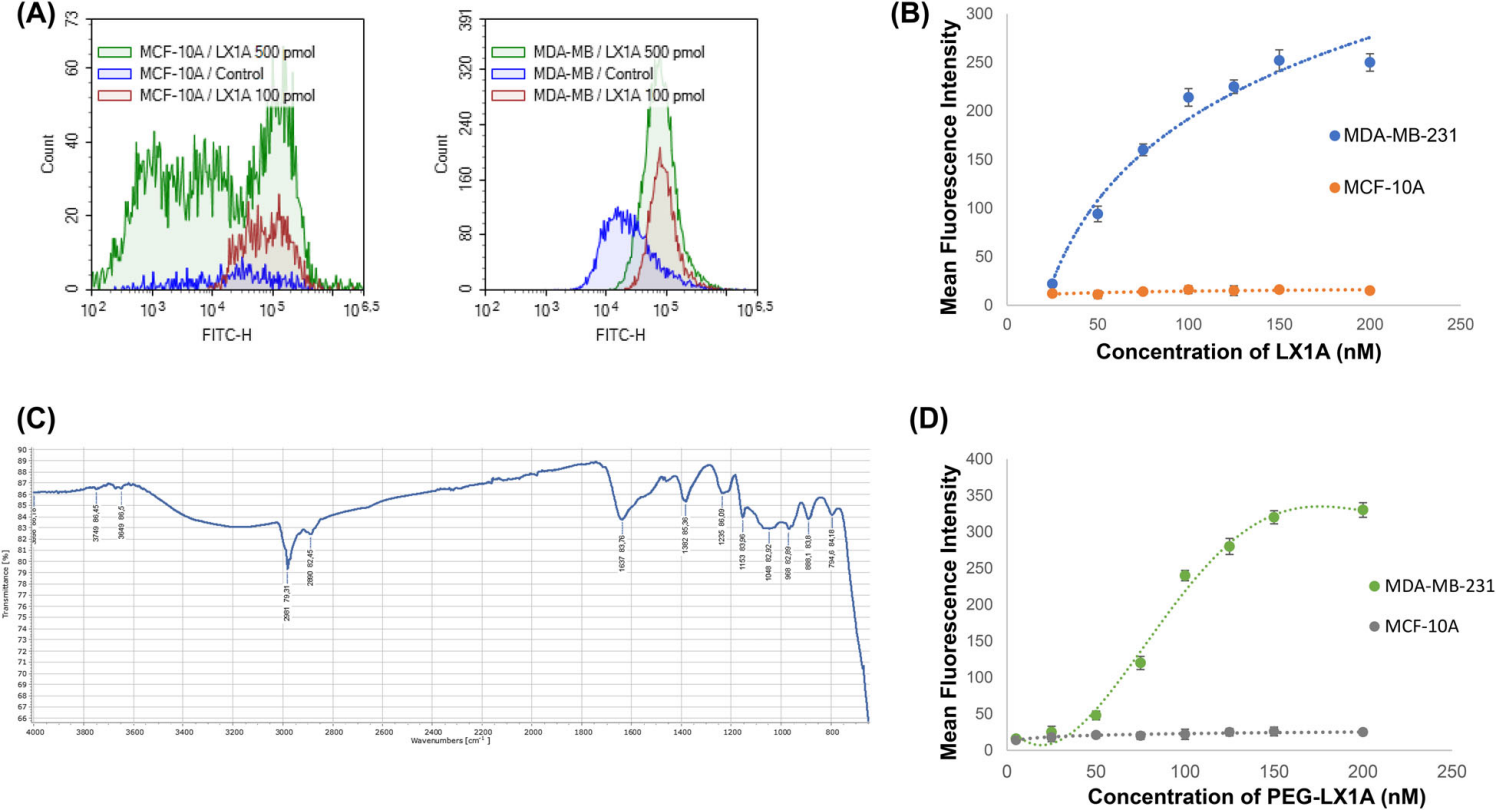
Binding of LX1A aptamer and its selectivity in MCF‐10A and MDA‐MB‐231 cells and KD curve analysis. (A) Flow cytometry binding graphs of aptamers in MCF‐10A and MDA‐MB‐231 cells (B) Concentration‐dependent binding kinetics of LX1A aptamer in cells. (C) FTIR analysis after PEG‐Aptamer conjugation. (D) Concentration‐dependent binding kinetics of PEG‐LX1A in cells. The data are presented as the mean ± SD from three independent experiments.

Binding of the LX1A aptamer in the control cell MCF‐10A breast cancer cells was not specific and no change in peak was observed as a function of concentration. However, it was found that the binding of LX1A aptamer placed in 100 and 500 pmol medium increased in MDA‐MB‐231 breast cancer cells and the peak shift could provide a clear binding (Fig. 1A). The relative Kd value was calculated as 62.7 ± 2.5 for the LX1A aptamer (Fig. 1D) and 84.4 ± 4.6 for the PEG‐LX1A aptamer (Fig. 1D).

When the FTIR spectrum of apt‐PEG binding (Fig. 1C) was examined, bands at 3749, 3649, 2981, 2890, 1637, 1382, 1235, 1153, 1048, 968, 888, and 794 cm^−1^ were obtained. The vibrations of carbonyl bond (C=O) formed as a result of PEG‐aptamer binding are observed at 1637 cm−1, and the band belonging to C—N vibrations is observed at 1382 cm^−1^ [24, 25] C—H stretching vibrations were obtained at 2981 cm−1, and C—H bending vibrations were obtained at 1235 cm−1. The bands at 1048 and 1153 cm−1 belong to C—O stretching vibrations [26].

### 3.2 Characterization of TRAIL‐loaded PLGA nanoparticles

The solubility of the TRAIL protein in PBS pH 7.4 buffer was calculated to be 5.163 mg·mL^−1^ based on UV measurements. The characterization results of the formulation improvement studies not loaded with TRAIL are presented as mean ± standard deviation in Table S3. As an indicator of the reliability of the measurements, the reference value (baseline) should ideally be 1000 and the ideal value of the intercept of the correlation function (g2; intercept) should be between 0.85 and 0.95. According to the measurement results, the reference value (baseline) of all samples varied between 0.992 and 1.229. The intercept of the samples varied between 0.8577 and 0.9261 and was found to be in the ideal range.

**Fig. 2:**
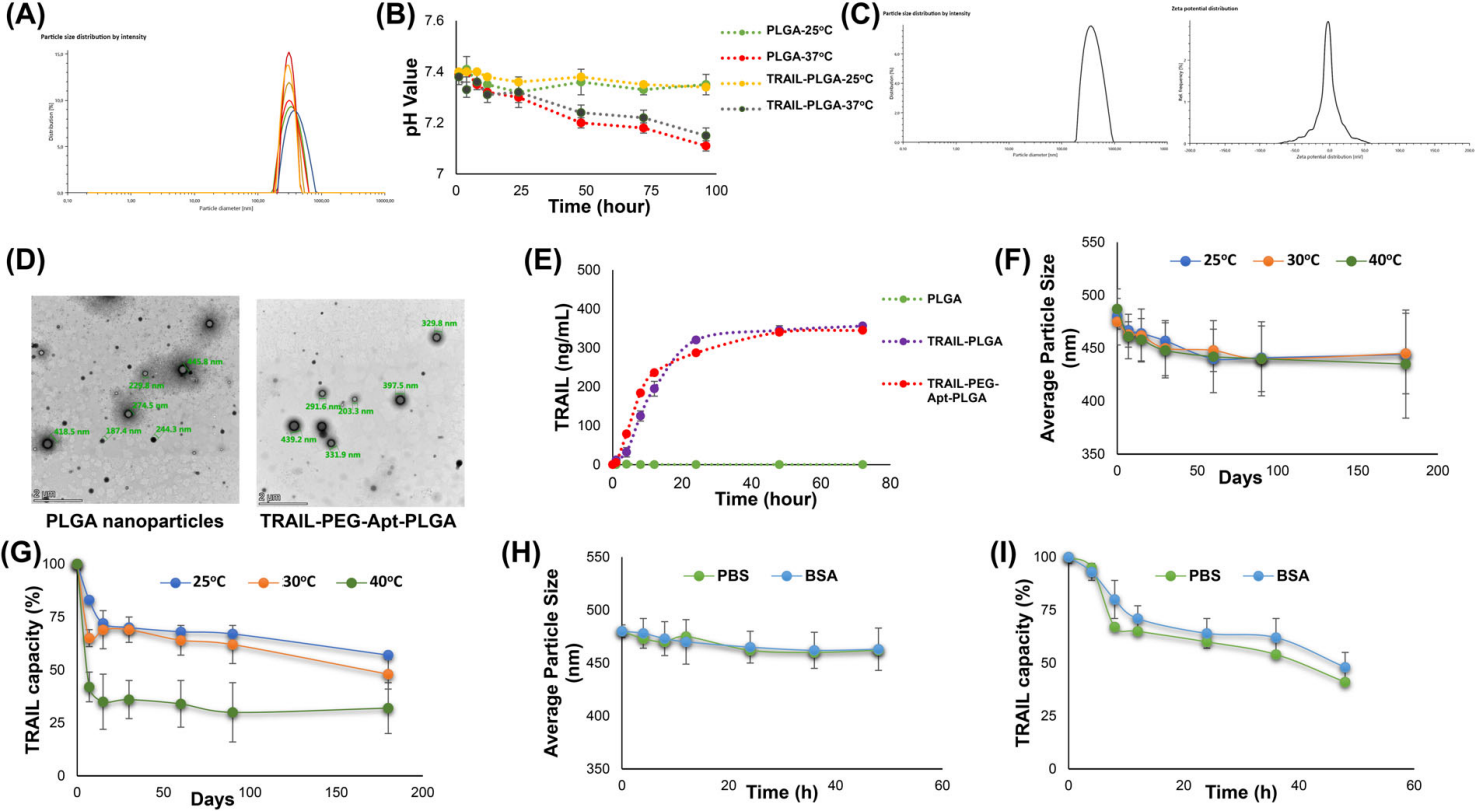
TRAIL‐loaded PLGA nanoparticles characterization analysis and investigation of formulation depending on time and days. (A) Particle size results in zeta potential determination. (B) pH changes in TRAIL‐loaded and unloaded PLGA at 25 °C and 37 °C. (C) Particle size and zeta charge graph of TRAIL‐loaded PEG‐Apt‐PLGA nanoparticles. (D) TEM images of PLGA and TRAIL‐loaded PEG‐Apt‐PLGA nanoparticles (Scale bar 2 μm). (E) Time‐dependent (0–72 h) TRAIL release profiles from TRAIL‐loaded PLGA and TRAIL‐loaded PEG‐Apt‐PLGA nanoparticles. (F) Average particle size changes of TRAIL‐PEG‐Apt‐PLGA nanoparticles at 25 °C, 30 °C, and 40 °C during 180‐day stability testing. (G) TRAIL capacity changes of TRAIL‐PEG‐Apt‐PLGA nanoparticles at 25 °C, 30 °C, and 40 °C during 180‐day stability testing. (H) Average particle size changes of TRAIL‐PEG‐Apt‐PLGA nanoparticles in PBS or BSA at 37 °C during 48 h stability testing. (I) TRAIL capacity changes of TRAIL‐PEG‐Apt‐PLGA nanoparticles in PBS or BSA at 37 °C during 48 h stability testing. The data are presented as the mean ± SD from three independent experiments.

When the results of the particle size (Fig. 2A) measurements were evaluated, it was seen that the particle size increased when the amount of PVA in the formulation exceeded 100 mg. When the amount of PVA was increased to 200 mg, the particle size increased to 400 nm, and when it was increased to 500 mg, it exceeded 500 nm. The fact that the amount of PVA was less than 100 mg had no direct effect on the particle size. Increasing the amount of PLGA caused the particle size to decrease. Therefore, the most appropriate amount of PLGA was determined to be 25 mg. In addition, reducing the probe sonication intensity and duration increased the particle size. The particle size increased more than 4 times with the decrease in intensity or duration.

The polydispersity index is an important parameter that shows the particle size distribution and homogeneity. A polydispersity index higher than 0.7 indicates that the distribution is not homogeneous. The polydispersity index of the samples increased as the amount of PVA increased, but it varied between 0.057 and 0.297, and all values were found below 0.7. The measurements showed that all the samples had homogeneous distribution.

The zeta potential value is an important indicator of colloidal stability and product quality. In general, samples with zeta potential values greater than ±30 mV are considered to be highly stable. The zeta potential values of the samples varied between −21.7 and −4.7 mV. Although the zeta potential value moved away from zero as the PVA increased, this required the use of relatively large amounts of PVA. In this situation, the particle size increased significantly.

Considering the particle size, polydispersity index and zeta potential values as a whole, the most ideal formulations were determined as formulations coded F1, F4 and F11. The formulation contents and results are shown in Table S4.

The values of the intersection point of the TRAIL‐loaded formulations ranged from 0.8870 to 0.9210, and these values are between the ideal value range of the intersection point, 0.85–0.95. The baseline values ranged between 1.002 and 1.031, which is very close to the ideal reference value of 1000. The results obtained showed that the measurements were reliable. Particle sizes ranged from 343.0 nm to 410.2 nm, and it was observed that particle size increased with TRAIL loading. While an average increase of 65 nm was observed for the 1 T and 4 T formulations, this increase was found to be slightly higher for the 11 T formulation (130 nm). A similar increase was observed in the polydispersity index, but the results were still within ideal limits. The zeta potential values were slightly closer to zero. Although the zeta potential value is not a direct indicator of particle surface charge, it can be considered as an indication that the TRAIL protein is attached to the surface. The 11 T formulation was found to be suitable in terms of loading and size and was attached with PEG‐Apt (Table S5).

### 3.3 Dissolution studies and loading capacity results

To determine whether the lactic acid and glycolic acid released during the degradation of PLGA in the 11 T formulation could affect the pH, pH changes were measured. When TRAIL‐loaded and unloaded PLGA was measured with a pH meter in pH 7.4 PBS buffer at 25 °C and 37 °C for 96 h, the pH changes did not show any significant changes Fig. 2B. When the zeta potential of TRAIL‐loaded PEG‐aptamer PLGA nanoparticles was investigated, the particle size was determined to be 473.4 ± 18.36, the polydispersity index was 0.218 ± 0.061, and the zeta potential value was −6.3 ± 0.2 mV (Fig. 2C). In the TEM electron microscopy analyses, it is predicted that the size of the spherical nanoparticles in TRAIL‐loaded PEG‐Apt‐PLGA nanoparticles increases and branching structures around the PLGA increase. This indicates that the binding is on the particle surface and that the spherical shape is stable with TRAIL loading and there is no structural change (Fig. 2D). It can be seen that the release results accelerate between 8 and 24 h and the amount of TRAIL increases in both nanoparticles. TRAIL release was found to be 80.05 ± 6.14% in TRAIL‐PLGA nanoparticles and 71.25 ± 4.62% in TRAIL‐PEG‐Apt‐PLGA nanoparticles in 24 h (Fig. 2E). Long‐term stability testing (180 days) was performed in PBS at 25 °C, 30 °C and 40 °C and accelerated stability testing was performed in BSA at 37 °C. In stability tests performed in PBS at 25 °C, 30 °C and 40 °C, the formulation showed a decrease in particle size up to 180 days (Fig. 2F) and this was achieved with TRAIL release (Fig. 2G). The graph (Fig. 2G) indicates that the degree of decrease intensified with increasing temperature. Since PLGA is not a temperature‐sensitive polymer, the observed increase in TRAIL levels at higher temperatures was attributed to the enhanced solubility of TRAIL. Given that the initial decrease appeared to be related to storage conditions (temperature), the findings suggest that storage of the formulation at refrigerator temperature may provide more favorable stability. In BSA stabilization studies performed at 37 °C, it was observed that the average particle size and TRAIL capacity were similar to PBS for 48 h (Fig. 2H,I).

### 3.4 Evaluation of TRAIL‐PEG‐apt‐PLGA formulation in MDA‐MB‐231 cells

**Fig. 3:**
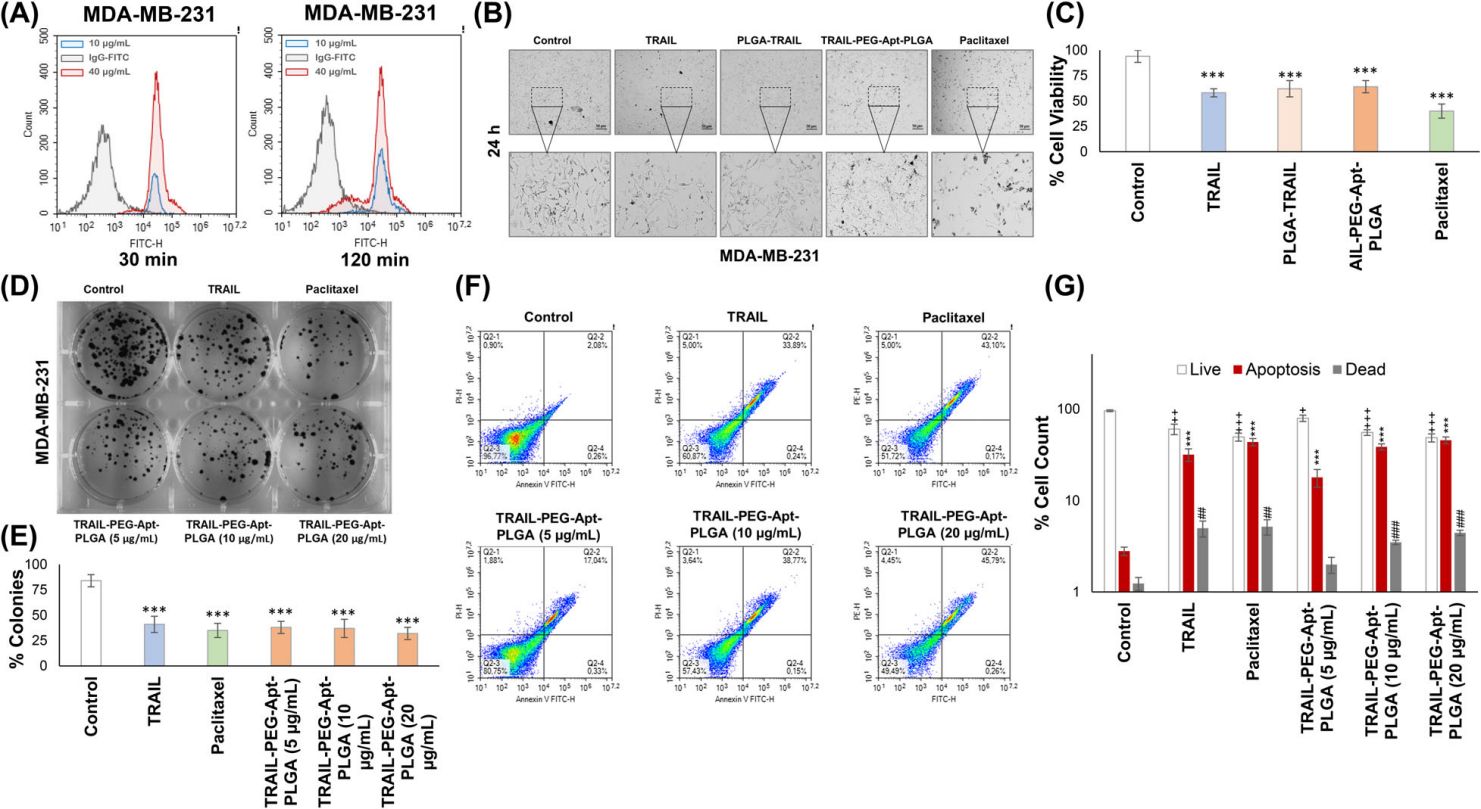
Cellular response of MDA‐MB‐231 cells to TRAIL‐based formulations and paclitaxel. (A) Binding analysis of TRAIL‐PEG‐Apt‐PLGA (10 and 40 μg·mL^−1^) at 30 and 120 min by flow cytometry. (B) Cellular morphology of comparative treatments in MDA‐MB‐231 cells with TRAIL (10 ng·mL^−1^), PLGA‐TRAIL (10 μg·mL^−1^), TRAIL‐PEG‐Apt‐PLGA (10 μg·mL^−1^), and paclitaxel (10 nm) for 24 h and (Scale bar 50 μm) (C) Cell viability following 24 h treatment (***P < 0.001 compared to control group). (D) Colony formation assay of MDA‐MB‐231 cells treated with TRAIL (10 ng·mL^−1^), TRAIL‐PEG‐Apt‐PLGA (5, 10, and 20 μg·mL^−1^), and paclitaxel (10 nm) for 14 days and (E) quantification of colony formation (***P < 0.001 compared to control group). (F) Annexin‐V binding graphics with flow cytometric analysis and (G) quantification of apoptotic cell percentages in MDA‐MB‐231 cells following 24 h treatment with TRAIL (10 ng·mL^−1^), PLGA‐TRAIL (10 μg·mL^−1^), TRAIL‐PEG‐Apt‐PLGA (10 μg·mL^−1^), and paclitaxel (10 nm). The data are presented as the mean ± SD from three independent experiments. The statistical significance were determined using Mann–Whitney U‐test (C,E,G). (^+++^ *P* < 0.001, ^++^ *P* < 0.01, ^+^ *P* < 0.05 compared to control group live cell; ***P < 0.001 compared to control group apoptotic cell; ^###^ *P* < 0.001, ^##^ *P* < 0.01 compared to control group dead cell).

It was ascertained that the TRAIL‐PEG‐Apt‐PLGA formulation, when added to the medium at concentrations of 10 μg·mL^−1^ and 40 μg·mL^−1^, exhibited binding to the surface of MDA‐MB‐231 cells at 30 and 120 min (Fig. 3A). The binding increased most significantly at 120 min, contingent on the concentration. The 24‐h incubation values were employed in the calculation of IC50 doses. During formulation development, PLGA, PEG‐PLGA, PEG‐Apt, and PEG‐Apt‐PLGA nanosystems were obtained. These nanosystems showed no cellular effects in cytotoxicity studies at a concentration of 10 μg·mL^−1^ (Fig. S2). Since they exhibited similarities to control cells, they were not used in subsequent experimental studies. The IC50 values for TRAIL, PLGA‐TRAIL, TRAIL‐PEG‐Apt‐PLGA, and paclitaxel were determined to be 8.165 ± 0.36 ng·mL^−1^, 12.7 ± 0.81 μg·mL^−1^, 11.7 ± 1.04 μg·mL^−1^, and 13.6 ± 2.21 nm, respectively. Paclitaxel was utilized as a positive control. TRAIL (10 ng·mL^−1^), PLGA‐TRAIL (10 μg·mL^−1^), TRAIL‐PEG‐Apt‐PLGA (10 μg·mL^−1^), and paclitaxel (10 nm) were introduced to MDA‐MB‐231 cells, and cell morphology (Fig. 3B) and viability (Fig. 3C) were assessed over a 24‐h period. Additionally, images and viability were examined at 12 and 48 h (Fig. S3). The study concluded that cell viability decreased with TRAIL‐PEG‐Apt‐PLGA, depending on the time point. The developed system exhibited a similar effect with TRAIL in *in vitro* studies. This effect was attributed to the fact that the protein's activity was preserved, the formulation was highly biocompatible, and it did not disrupt the protein's mechanism of action (*P* < 0.05, *P* < 0.01, *P* < 0.001).

A subsequent examination of the colony numbers at the conclusion of the 14th day reveals a dose‐dependent reduction in cells, attributable to the application of TRAIL and TRAIL‐PEG‐Apt‐PLGA formulations (Fig. 3D). While the colony numbers are reduced by half in some groups, they are reduced more than the control group in other groups (Fig. 3E, *P* < 0.001). This outcome can be attributed to the fact that the colonization percentages decrease in proportion to the increase in the amount of TRAIL released into the medium. Following CV staining, microscopic images were obtained to assess colony formation and cellular interaction of TRAIL‐PEG‐Apt‐PLGA at concentrations of 5, 10, and 20 μg·mL^−1^ (Fig. S4).

The binding percentages of Annexin‐V/PI in cells were subsequently calculated, revealing an increase in apoptotic cells in a dose‐dependent manner following the application of TRAIL and TRAIL‐PEG‐Apt‐PLGA formulations (Fig. 3F, *P* < 0.001). While the rate in the control group cells was approximately 2%, it was determined to be approximately 45% in high doses of TRAIL‐PEG‐Apt‐PLGA application (Fig. 3G).

### 3.5 Effect of TRAIL‐PEG‐apt‐PLGA formulation on breast tumor mouse model

**Fig. 4:**
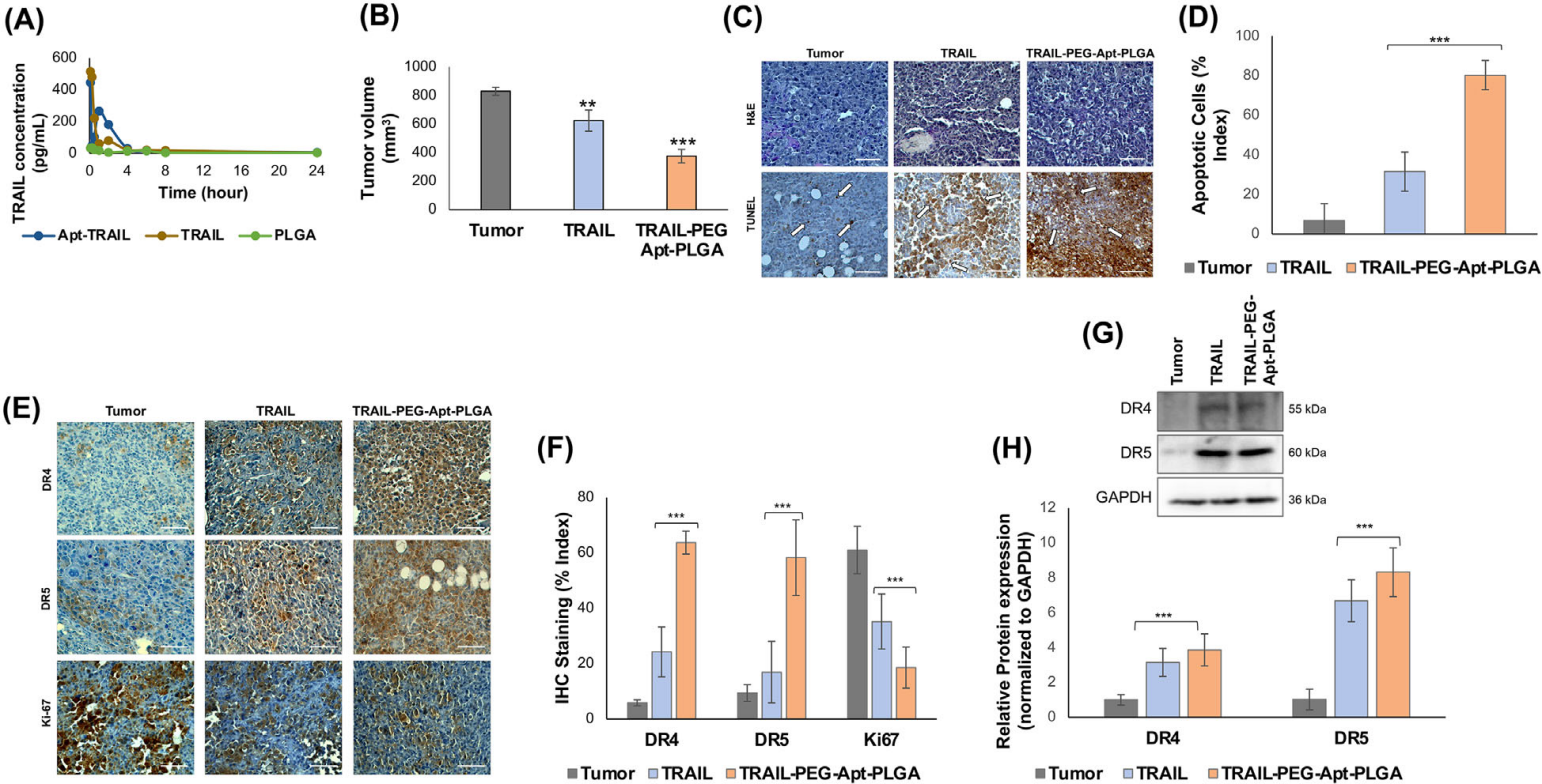
*In vivo* pharmacokinetic and tumor model studies of TRAIL‐PEG‐Apt‐PLGA nanoparticles in experimental animals, including tissue histological analyses. (A) Pharmacokinetic profile of plasma TRAIL concentration (pg·mL^−1^) during 24 h in BALB/c mice following administration of TRAIL (100 ng·kg^−1^) or TRAIL‐PEG‐Apt‐PLGA (100 μg·kg^−1^). Tumor response and molecular analyses in NOD/SCID gamma mice treated with TRAIL (100 ng·kg^−1^) or TRAIL‐PEG‐Apt‐PLGA (100 μg·kg^−1^). (B) Tumor volume measurements. (C) Histological analysis of tumor tissues using H&E and TUNEL staining (Scale bar 50 μm). (D) Quantification of apoptotic cell ratio via TUNEL staining (E) IHC staining of DR4, DR5, and Ki67 (Scale bar 50 μm). (F) Percentage indices of IHC staining in tumor tissue. (G) Western blot analysis of DR4 and DR5 protein expression and (H) quantification of protein expression differences between groups on tumor tissue. The data are presented as the mean ± SD from three independent experiments. The statistical significance was determined using Mann–Whitney *U*‐test (B,D,F,H). (***P < 0.001, **P < 0.01 compared to tumor group).

For the purpose of conducting pharmacokinetic studies, a single dose of TRAIL‐PEG‐Apt‐PLGA and TRAIL was administered to Balb‐c mice. The time to reach maximum plasma concentration (*t*_max_) was subsequently determined. For TRAIL, tmax was found to be 0.5 h, while for TRAIL‐PEG‐Apt‐PLGA, it was 4 h. The maximum plasma concentration (*C*_max_) was determined to be 480 ± 25 pg·mL^−1^ for TRAIL and 515 ± 36 pg·mL^−1^ for TRAIL‐PEG‐Apt‐PLGA. The area under the curve (AUC) from zero to 24 h (AUC0‐24, expressed in pg/mL*h) was determined to be 1124 ± 20 for TRAIL and 3914 ± 42 for TRAIL‐PEG‐Apt‐PLGA (Fig. 4A).

When the tumor sizes of the tumors developed in NGS mice were measured after treatment, they were determined to be 830 ± 68 mm^3^ in the tumor group, 642 ± 74 mm^3^ in the TRAIL group, and 575 ± 47 mm^3^ in the TRAIL‐PEG‐Apt‐PLGA group (Fig. 4B, *P* < 0.01, *P* < 0.001). At the same time, the total weights and tumor tissue weights of the mice were measured. There was no difference in total weights between the groups, but tumor weights were significantly reduced in the TRAIL and TRAIL‐PEG‐Apt‐PLGA groups (Fig. S5, *P* < 0.05, *P* < 0.01).

Histopathologic examination of mouse tumor tissues revealed a high‐grade epithelial tumor morphology. These neoplastic cells were observed to have extensive eosinophilic cytoplasm with indistinct borders. It was noted that mitotic structures between the cells increased as a result of tumor formation. It was observed that the number of necrotic cells increased compared to the number of tumor cells in TRAIL and Apt‐TRAIL (TRAIL‐PEG‐Apt‐PLGA) treatments (Fig. 4C). In tunnel staining, it was observed that the staining was more intense in Apt‐TRAIL (TRAIL‐PEG‐Apt‐PLGA) treated mice and the number of apoptotic cells significantly increased compared to the tumor group (*P* < 0.001). Similarly, the tumor tissue of TRAIL‐treated mice was more intense compared to the tumor group, and an increase in the number of apoptotic cells was observed (Fig. 4D, *P* < 0.01). It was determined that DR4 and DR5 expression increased in tumor tissues of mice with IHC (Fig. 4E,F, *P* < 0.001) that received TRAIL and TRAIL‐PEG‐Apt‐PLGA. Quantification of changes in IHC staining was compared with western blot (Fig. 4G). Subsequent to the western blot analysis of DR4 and DR5 protein expression changes, an increase was observed in the TRAIL and TRAIL‐PEG‐Apt‐PLGA groups in comparison to the tumor group. This increase was found to be statistically significant (Fig. 4H, *P* < 0.001). It was investigated whether the formulations applied to the tumor‐infected animals resulted in a change in the treatment of the secondary organs. In the experiments conducted, HE staining was performed on the lung, liver, and kidney tissues of the mammary tumor‐bearing mice and examined. No changes were observed in the lung, liver, and kidney tissues in the evaluations (Fig. S6).

### 3.6 RT^2^ profiler PCR array results

**Fig. 5:**
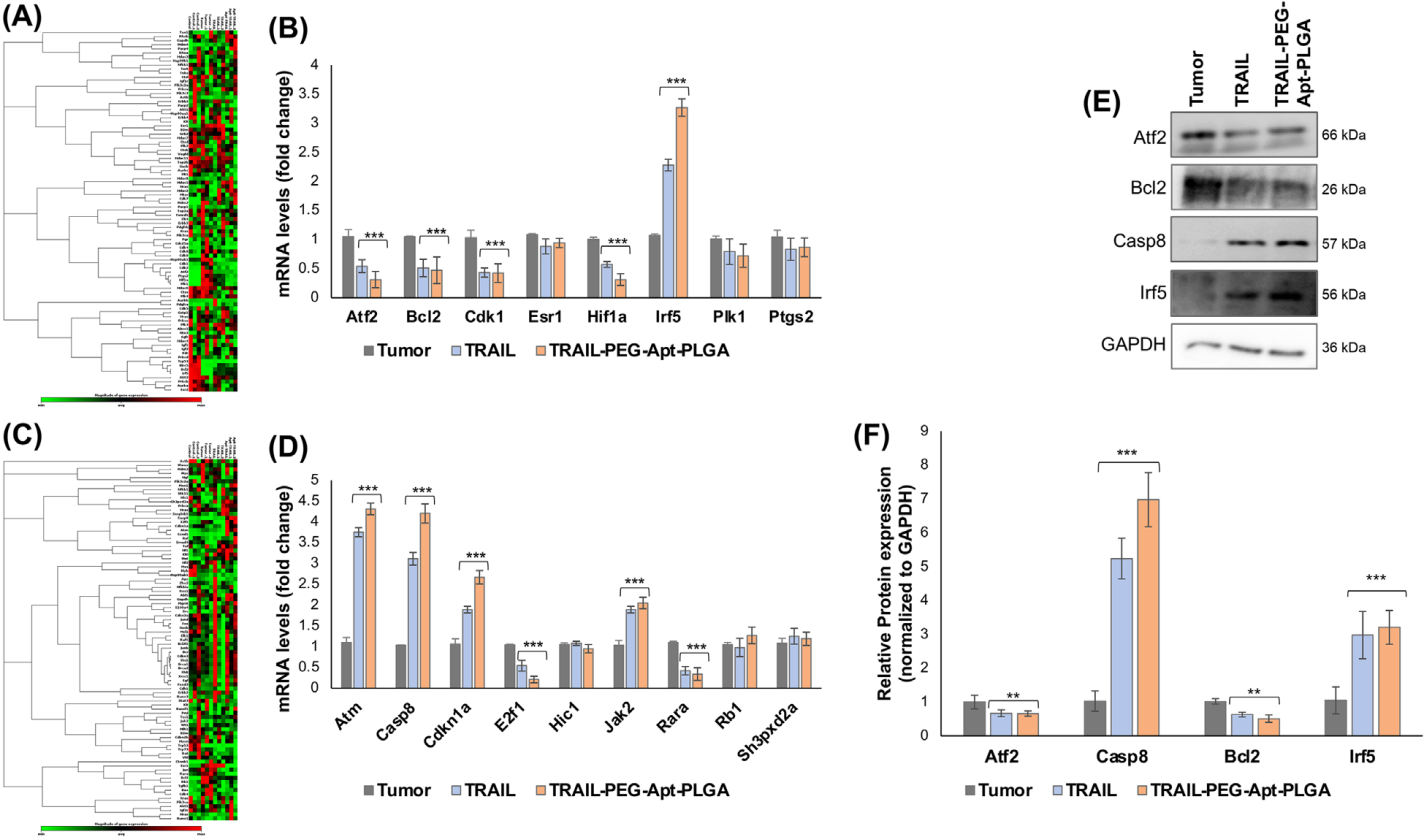
PCR Array analysis of tumor tissues from NOD/SCID gamma mice treated with TRAIL (100 ng·kg^−1^) or TRAIL‐PEG‐Apt‐PLGA (100 μg·kg^−1^). (A) Cancer Drug Target RT^2^ Profiler PCR Array Clustergram and (B) Comparison of Atf2, Bcl2, Cdkn1, Esr1, Hif1a, Irf5, Plk1, Ptgs2 mRNA levels between groups in Cancer Drug Target (C) Oncogene Tumor Suppressor RT2 Profiler PCR Array Clustergram analyses and (D) Comparison of Atm, Casp8, Cdkn1a, E2f1, Hic1, Jak2, Rar, Rb1, Sh3pxd2a mRNA levels between groups in Oncogene Tumor Suppressor (E) Western blot bands of Atf2, Casp8, Bcl2, Irf5 protein expression levels and (F) comparison graphs between groups. The data are presented as the mean ± SD from three independent experiments. The statistical significance was determined using Mann–Whitney *U*‐test (B, D, F). (***P < 0.001, **P < 0.01 compared to tumor group). Aptamers are used both therapeutically and as targeting agents in cancer treatment. We developed an aptamer‐targeted PLGA–TRAIL nanosystem that exhibited superior therapeutic efficacy in NOD/SCID breast cancer models. This nanosystem represents a novel biotechnological drug candidate for suppressing resistance development in breast cancer.

In Cancer Drug Target RT^2^ Profiler PCR Array Analyses (Fig. 5A), an increase in Atf2, Bcl2, Cdk1, Cdk2, Cdk8, Esr1, Hif1a, Mdm2, Plk1, Ptgs2 genes was observed in the tumor group when compared with the control group. Conversely, Birc5, Irf5, Trp53 genes were suppressed, which was found to be statistically significant (*P* < 0.0001–*P* < 0.05). A comparison of the TRAIL treatment group with the tumor group revealed an increase in Irf5 genes, while Atf2, Bcl2, Cdk1, Hif1a, and Plk1 genes were suppressed. This finding was found to be statistically significant (*P* < 0.0001–*P* < 0.05). In the TRAIL‐PEG‐Apt‐PLGA group, an increase in Irf5 genes was observed, while Atf2, Bcl2, Cdk1, Esr1, Hif1a, Plk1, and Ptgs2 genes were suppressed, and this difference was found to be statistically significant (*P* < 0.0001–*P* < 0.05). Conversely, when the TRAIL‐PEG‐Apt‐PLGA group was compared with the tumor group, no increase in genes was observed, while a suppression in Esr1, Hif1a, Plk1, Ptgs2 genes was found to be statistically significant (*P* < 0.0001–*P* < 0.05) (Fig. S7A,B).

Following the analysis of the genes exhibiting differences between the groups in the Oncogene Tumor Suppressor RT^2^ Profiler PCR Array Analysis (Fig. 5C), genes demonstrating 2‐fold or greater change and achieving a significance level less than *P* < 0.05 were identified. A subsequent comparison of the tumor group with the control group revealed an increase in the Atm, Bcl2, E2f1, Esr1, Met, Rara, Rb1, Tgfb1 genes, while the Hic1, Mcl1, Trp53 genes were suppressed. This finding was found to be statistically significant (*P* < 0.0001‐*P* < 0.05). In the TRAIL treatment group, an increase was observed in Casp8 and Jak2 genes, while Bcl2, E2f1, Rara genes were suppressed, which was found to be statistically significant (*P* < 0.0001‐*P* < 0.05). In the TRAIL‐PEG‐Apt‐PLGA group, an increase was observed in Atm, Casp8, Sh3pxd2a genes, while Esr1, Rara, Rb1 genes were suppressed, and this was found to be statistically significant (*P* < 0.0001‐*P* < 0.05). In addition, when the TRAIL‐PEG‐Apt‐PLGA group was compared with the tumor group, an increase was observed in Atm, Casp8, Cdkn1a, E2f1 genes, while low suppression was observed in Esr1, Rara genes. This finding was also found to be statistically significant (*P* < 0.0001‐*P* < 0.05) (Fig. S8A,B).

A comparative analysis was conducted between the TRAIL and TRAIL‐PEG‐Apt‐PLGA treatment groups and the tumor group. The expression levels of select genes, including Atm, Casp8, Cdkn1a, Irf5, and Jak2, were elevated, while Atf2, Bcl2, Cdk1, E2f1, Esr1, Hif1a, and Rara exhibited a decrease in expression. These alterations were quantitatively assessed through qPCR and were found to be statistically significant (*P* < 0.001). No significant alterations were detected in the Hic1, Plk1, Rb1, Ptsg2, and Sh3pxd2a genes between the groups (Fig. 5B,D).

Genes that demonstrated alterations and were associated with TRAIL were quantitatively assessed and analyzed using western blot to ascertain if there was a discrepancy in protein expression (Fig. 5E). The analysis revealed alterations in the protein expressions of Atf2, Casp8, Bcl‐2, and Irf5. The application of TRAIL and TRAIL‐PEG‐Apt‐PLGA led to an augmentation in Casp8 (*P* < 0.001) and Irf5 (*P* < 0.001) expression levels in breast tumors, accompanied by a suppression of Atf2 (*P* < 0.01) and Bcl‐2 (*P* < 0.01) protein expressions. These alterations were found to be statistically significant (Fig. 5F).

## 4 Discussion

In the context of drug loading studies, the selection of an appropriate carrier is of paramount importance. The carrier system must be of an appropriate size for the targeted activity, capable of dissolving the drug, and capable of holding it in sufficient quantities to release it in the desired region. In addition to the physicochemical properties of the drug molecule, such as particle size and/or molecular weight, solubility, and surface charge, the compatibility and affinity between the drug and the carrier system are critical factors in determining the reliability and effectiveness of the drug carrier system complex. The affinity between the carrier system and the drug also affects drug loading capacity and release. For instance, while the addition of surfactant has been demonstrated to modify loading capacity and release, lipophilic drugs generally exhibit heightened loading capacity and prolonged release within lipid structures. The route of administration is another salient parameter that influences the selection of the carrier system [27, 28, 29].

In the context of the study, TRAIL protein‐loaded PLGA nanoparticles will be fabricated, and the nanoparticles will be functionalized with the PLGA‐PEG‐aptamer combined structure [20]. The PLGA nanoparticles were fabricated using the solvent evaporation method. The rationale for employing PLGA nanoparticles stems from the fact that both the TRAIL protein and the aptamer structure exhibit high water solubility. This choice ensures that the interaction of highly water‐soluble active substances with the carrier system is minimal when administered in oil‐based carrier systems, such as liposomes and solid lipid nanoparticles. As the aptamer and TRAIL molecule utilized in the study were situated in the aqueous phase, their encapsulation capacities remained uncompromised. This ensured that there was no competition for solubility between the two molecules within the limited space available. The potential for an increase in particle size through further studies aimed at enhancing encapsulation capacities is also a promising avenue for exploration. However, an increase in particle size may present challenges for intravenous application and could potentially enhance the susceptibility to phagocytosis within the blood circulation system, potentially compromising the targeted delivery to the intended area. Furthermore, the size of the particles directly correlates with their release capacity, cell internalization, and passive targeting. In the *in vivo* findings, the particle size was not sufficiently large to exceed this limit. In the pharmacokinetic studies in mice, it was determined that the particle size could be released in circulation and did not affect internalization. The employment of a water‐based drug carrier system has been demonstrated to facilitate TRAIL's delivery to the intended location and ensure its expeditious release in *in vivo* applications. The TRAIL and aptamer were bound to the same region in the water‐based drug carrier system as a monolayer. In this configuration, competitive solubility was achieved, and the binding rates of TRAIL and the aptamer exhibited variation. The aptamer exhibited selectivity for the target region, while TRAIL's release was contingent upon binding to the receptor. Furthermore, TRAIL demonstrated a stable structure during circulation. Furthermore, in these systems, TRAIL protein can bind to the aptamer instead of binding directly to the structure.

In order to ascertain the most efficacious configuration in the prepared system, it was determined that the structure underwent the following three steps sequentially. It was demonstrated that the structure exhibited durability until it was delivered to the target tissue in the blood circulation system. Upon reaching the target tissue, the structure recognized the receptors, interacted with these receptors, and established a tight connection. The TRAIL effective molecule was then released from the structure attached to the cell receptors.

The absence of interaction between the aptamer, which has an affinity for a specific receptor, and the TRAIL molecule, which is known to have anticancer activity, enabled the aptamer to fully fit into the domain outside the cell and establish a tight connection. The carrier system enabled the release of the TRAIL molecule, which then interacted with the aptamer at a minimal level [30]. This interaction occurred outside the cell, resulting in the binding of the TRAIL molecule to the DR5 and DR4 receptors and the subsequent activation of the apoptotic pathway. The absence of DR4/DR5 on the surface of cancer cells makes them resistant to TRAIL‐induced apoptosis, regardless of the status of other apoptosis signaling components [31, 32]. In fact, TRAIL release stimulates DR4 and DR5 receptors in cells, which accelerates the apoptotic signal in cancer cells. An increase in the number of receptors then contributes to increased TRAIL binding and accelerates the tumor‐shrinking effect. Studies suggest that increased DR4 and DR5 expression when TRAIL binds to the receptor is due to the receptor complex entering the endosome and creating stress in the endoplasmic reticulum. This stress activates transcription factors, accelerating DR4 and DR5 expression [33]. This study suggests that increased DR4/DR5 expression can overcome TRAIL resistance and that targeted TRAIL binding may be effective in treating TNBC. A carrier system comprising two distinct liquid layers was utilized in the study, with the objective of producing nanoparticles in the form of a water/oil/water (w/o/w) type emulsion. The aqueous layer, situated within the innermost phase, is where the primary activity responsible for delivering the TRAIL protein is expected to occur. The oil layer, positioned centrally, consists of a PLGA copolymer. The aqueous layers and the aqueous layer in the outermost phase are designed to carry the PEG‐aptamer complex. The hydrophilic structure of the outermost layer enabled intravenous administration, and the PEG polymer in this layer prevented the system from being recognized by the RES and discarded [34]. A survey of the extant literature reveals analogous studies on TRAIL loading onto PLGA nanospheres employing the solvent evaporation method. These studies have reported loading efficiencies ranging from 40% to 90% [19, 35].

The intravenous injection of developed antineoplastic drugs into tumor tissue is quite restrictive, especially when the objective is to provide tumor shrinkage by targeting the desired tissue. The injection of antineoplastic drugs into the tumor site poses a significant challenge in clinical applications. Consequently, the objective of this study was to develop a nanoformulation capable of delivering targeted therapy and inducing tumor shrinkage. A PLGA microsphere formulation developed to provide long‐term release of TRAIL was shown to be very effective *in vivo* and to shrink the tumor when injected into the close region of the tumor in a tumor model created with Hela cells [36]. In a separate study, a TRAIL‐loaded analog was developed using PEG with varying molecular weights (2, 5, 10, 20, 30 kD) and evaluated in a tumor model created using HCT116 colon cancer cells. It has been reported that the 30 K‐PEG‐TRAIL analog releases more TRAIL within 5 days and causes significant shrinkage in tumor tissue [15]. While TRAIL has been observed to develop resistance in tumor tissue after a certain period, this resistance has not been observed when its release is regulated with different formulations. Consequently, the development of formulations with varied structures has been proposed as a strategy to enhance TRAIL's efficacy. This study has yielded a solution to address this deficiency.

A study was conducted with colorectal adenocarcinoma cells (COLO 205) to examine the effects of the ES/TRAIL liposome complex on cancer metastasis and tumor growth. The results demonstrated that the complex hinders metastasis and suppresses the growth of tumor tissue. The study's methodology entailed the targeting of the receptor for E‐selectin (ES) leukocytes and the binding of recombinant TRAIL to this complex, which was then bound to TRAIL receptors on both ES and cancer cells. The study's findings, as reported by Mitchell *et al*. in 2014 [37], indicated that the injection of the complex, obtained after tumor formation in C57BL/6J mice, led to a reduction in tumor size within 2 weeks. The TRAIL complex was found to suppress tumor growth via leukocytes. It is noteworthy that TRAIL sensitivity exhibits variability among different cancer cell types; however, its efficacy is generally augmented by the concurrent administration of TRAIL and a chemotherapeutic agent. *In vivo* studies in murine tumor models have reported that TRAIL can show serious antitumor effects alone or in combination with chemotherapy or radiation therapy. These agents include doxorubicin, cisplatin, 5‐fluorouracil, paclitaxel, proteasome inhibitors, antibodies, and natural compounds [38]. Given the high toxicity of systemic chemotherapeutic agents, combination studies with TRAIL are prominent in cancer treatments, with the objective of increasing the effectiveness by using lower amounts of chemotherapeutic agents [39, 40].

In the tumor model established using A549 lung cancer cells and BALB/c mice, the efficacy of the liposome carrier system, loaded with DOX and TRAIL, on tumor growth was demonstrated both *in vivo* and *in vitro*. This study reported that the liposome‐mediated release in plasma could be controlled, providing a distinct advantage over their use in isolation. It was proposed that the cardiotoxic effects of DOX could be mitigated, and that the combination of low‐dose TRAIL with DOX was more efficacious [41]. In a subsequent study, the combination of TRAIL and Paclitaxel, bound to serum albumin‐bound nanoparticles, was investigated for its effectiveness in an *in vivo* tumor model. In the study, PTX and TRAIL/PTX nanoparticles were administered subcutaneously to BALB/c mice with pancreatic tumors for a period of 10 days. The study's findings, as reported by Min *et al*. in 2015 [42], indicated that the combination of TRAIL and PTX nanoparticles led to a significant reduction in tumor size. In a recent study, TRAIL and odanacatib (ODN) loaded PLGA nanoparticles (TRAIL‐ODN‐PLGA‐NPs) were administered to nude female mice in a kidney tumor model using Caki cancer cells. The results demonstrated that the tumor tissue in mice underwent a reduction in size, and *in vitro*, the nanoparticles exhibited an impact on mitochondrial dynamics via Bim protein stabilization in Caki cells, thereby inducing cell death by altering the raptor protein stabilizing Bim protein on regulation [43].

In the present study, it was ascertained that TRAIL and TRAIL‐PEG‐Apt‐PLGA formulations induced cell apoptosis in both *in vitro* and *in vivo* applications. In the context of apoptosis studies conducted on MDA‐MB‐231 cells, it was ascertained that TRAIL could be released from the formulation and could facilitate intracellular signaling by binding to receptors on the cell surface without compromising its efficacy. The experiments were conducted *in vitro*, under a short incubation period and a controlled cell culture environment (without plasma proteases or other degradation pathways). Under these conditions, TRAIL degradation is minimal; therefore, both free TRAIL and TRAIL‐PEG‐Apt‐PLGA exhibit comparable biological activity at the same concentration. The lack of plasma proteases and the relatively short incubation time (24 h) may explain why TRAIL shows similar *in vitro* activity to the TRAIL‐PEG‐Apt‐PLGA formulation. Nevertheless, *in vivo* studies have demonstrated that TRAIL formulated with the aptamer displays enhanced efficacy at both the gene expression and receptor activation levels. This improvement is likely due to the targeted localization of the aptamer‐conjugated formulation within tumor tissues. In the *in vivo* mouse tumor model, the intravenous application of the TRAIL‐PEG‐Apt‐PLGA formulation was found to be reliable and capable of readily identifying the tumor target with the aptamer, thereby providing signaling through binding to DR4 and DR5 receptors in the surrounding cells by releasing TRAIL. The *in vivo* study demonstrated the efficacy of the TRAIL‐PEG‐Apt‐PLGA formulation in reducing tumor size and modulating gene expression patterns, further substantiating its potential for utilization in breast cancer treatment. The observed alterations in biomolecules, including Atf2, Caps8, Bcl2, and Irf5, both at the gene and protein levels, substantiate the intramolecular interactions within the cell that are initiated by TRAIL.

The activation of transcription factor 2 (Atf2) is crucial for the execution of pivotal biological and cellular functions. It is a member of the leucine zipper family of DNA‐binding proteins, which are responsible for regulating various genes. Atf2's role in cellular proliferation and apoptosis is well‐documented; however, its precise function in breast cancer remains to be fully elucidated. While the role of Atf2 as an oncogene that promotes cell proliferation and worsens the outcome of cancers has been suggested, it is also assumed that Atf2 plays a tumor suppressor role in estrogen receptor positive breast cancer. In the present study, Atf2 expression levels were examined in tumor tissues and treatment groups. The results indicated that Atf2 expression was increased in tumor tissues and decreased in treatment groups. It has been demonstrated [44] that Atf2 increased the transcription of genes such as MMP13, cyclin A, aromatase, and MMP2, which may contribute to breast cancer metastasis and proliferation. The increased transcription of MMP13 by Atf2 may play a role in facilitating the metastasis of breast cancer to bone. Furthermore, it revealed that the transcription of cyclin A is predominantly governed by cJun‐Atf2 dimers, which have been demonstrated to enhance cell proliferation. Caspases involved in intracellular apoptosis events can be activated in a variety of ways. Caspase‐8 (Casp8) is a protein that is particularly activated by the effect of TRAIL and plays a role in extracellular apoptosis pathways. The interaction of TRAIL with death receptors (Death Receptors, DR4 and DR5) on the cell surface initiates a series of events, leading to the trimerization of receptors and the formation of DISC (Death‐Inducing Signaling Complex). Casp8, a constituent of this complex, undergoes activation within this process, thereby initiating the apoptotic response. The study revealed that the blockade of Casp8 is observed in cells that develop TRAIL resistance, and that when cells manage to overcome the death process, the tumor continues to develop [45]. In the present study, we demonstrated that the TRAIL‐PEG‐Apt‐PLGA formulation activates Casp8 at both the gene and protein levels through the release of TRAIL.

BCL2 is an anti‐apoptotic protein that helps cells resist apoptosis. This protein has been shown to suppress apoptotic signals within cells, thereby preventing the death of cancerous cells. Its expression, particularly in cancerous cells, has been associated with chemoresistance, radioresistance, and resistance to biological treatments. BCL2 functions by impeding the release of proapoptotic factors, such as cytochrome c, into the cytoplasm by reducing the permeability of the outer mitochondrial membrane [46, 47]. This, in turn, hinders the full activation of caspases, thereby impeding the process of TRAIL‐induced cell death. Our study revealed that Bcl‐2 was suppressed at both the gene and protein levels in tumor tissues using the TRAIL‐PEG‐Apt‐PLGA carrier system. The controlled release of TRAIL, as facilitated by the carrier system, proved instrumental in surmounting this resistance and effectively suppressing the anti‐apoptotic function of Bcl‐2.

IRF5, or Interferon Regulatory Factor 5, is a transcription factor that plays a critical role in regulating immune system responses. It has been demonstrated to play a pivotal role in the regulation of both innate and adaptive immune responses, as well as in antiviral defense. However, it is also associated with apoptosis and can induce apoptosis in various cellular stress conditions [48, 49]. It has been demonstrated that Irf5 can activate death receptor‐mediated apoptosis pathways, especially when stimulated with death ligands such as TNF‐alpha and TRAIL; Irf5 expression can increase and activate molecules that initiate apoptosis signaling (e.g., Cas8) [50]. Irf5 has been observed to enhance the transcription of various proapoptotic genes. It has been observed to direct cells towards apoptosis by increasing the expression of mitochondrial apoptotic factors such as BAX and PUMA. The activation of these genes has been shown to increase the permeability of the mitochondrial outer membrane, potentially triggering apoptosis via the mitochondrial pathway [51, 52]. In the context of the TRAIL‐PEG‐Apt‐PLGA carrier system, it has been observed that Irf5 levels in tumor tissues have been shown to increase both protein and gene expression, thereby regulating immune responses in conjunction with apoptotic mechanisms within the cells.

The phenomenon of TRAIL resistance can be attributed to various factors, including differential expression levels of pro‐ and anti‐apoptotic proteins in cells or microRNAs that can target proteins involved in the TRAIL pathway. In this case, it has been observed that the combination of TRAIL with conventional chemotherapy or radiotherapy can overcome the resistant phenotype, even when not administered alone. This suggests the need for the development of different formulations for TRAIL. One such formulation is dulanermin, a zinc‐coordinated preparation consisting of amino acids 114–281 of the extracellular domain of the TRAIL ligand [52, 53]. In preclinical studies, dulanermin has been demonstrated to selectively induce apoptosis in cancer cells while sparing most normal cells [54, 55]. It has demonstrated cytotoxic activity in various *in vitro* and *in vivo* models of solid and hematological malignancies, both as a monotherapy and in combination with chemotherapy [56, 57, 58, 59]. The initial human clinical trial (*n* = 71) conducted on patients with advanced cancer established the safety of dulanermin when administered as a monotherapy [60]. The combination of dulanermin with paclitaxel and carboplatin bevacizumab was reported to be well tolerated without increased toxicity in a previously untreated, advanced, non‐squamous NSCLC patient population (*n* = 24) [61]. Dulanermin functions by binding to DR4 and DR5; however, the expression of the receptor was not thoroughly examined in these studies. It is plausible that the expression of both receptors may be altered in advanced stages of tumors. Alternatively, tumor resistance may be augmented, and advanced cancer may also develop other mechanisms. Therefore, it is imperative to investigate the mechanisms of drugs targeting DR4 and DR5, such as TRAIL, in the cell and to demonstrate the pathways they can induce in tumors *in vivo*. The development of stable pharmaceuticals and the design of such medications to be released at the appropriate time once they have reached their target require the application of novel biotechnological drug approaches.

## 5 Conclusion

A biotechnological drug candidate has been developed for TRAIL, which has a targeted approach to breast cancer. The utilization of biotechnological drugs, exemplified by TRAIL‐PEG‐Apt‐PLGA, in the early stages of tumor development has the potential to circumvent TRAIL resistance. Furthermore, the combination of this drug with other medications holds promise for the treatment of various tumors in the future.

In summary, the findings of this study indicate that the TRAIL‐PEG‐Apt‐PLGA biotechnological drug candidate, which was developed in the study, is suitable for combined therapies. This suggests that further clinical studies may support the development of alternative applications for treating cancer patients.

## Conflict of interest

The authors declare no conflict of interest.

## Author contributions

GMD and OC conceived the study. OC supervised the study. OE, BK and TC performed recombinant protein production. AK, OE, TC and OC performed of cell culture studies. GDM and SO designed and performed animal experiments. AK and EG performed histological assessments of mouse tissue. GDM, EC and OC designed and performed the formulation studies. GDM and OC performed statistical analysis. GDM and OC wrote the manuscript with intellectual input from all authors.

## Supporting information


**Table S1.** PLGA nanoparticles (without TRAIL).
**Table S2.** PLGA nanoparticles (with TRAIL).
**Table S3.** Particle size, polydispersity index and zeta potential measurement results of PLGA nanoparticles (without TRAIL).
**Table S4.** Characterization results of ideal PLGA nanoparticles (without TRAIL).
**Table S5.** Characterization results of ideal PLGA nanoparticles (with TRAIL).

**Figure S1.** The efficiency of protein TRAIL purification and fractionation.
**Fig. S2.** Cell viability of TRAIL‐free nanosystems.
**Fig. S3.** Cellular viability of MDA‐MB‐231 cells to TRAIL‐based formulations and paclitaxel for 12 h and 48 h.
**Fig. S4.** Microscope images of CV staining.
**Fig. S5.** Total weight changes and tumor weights in in NOD/SCID gamma mice.
**Fig. S6.** HE staining images of lung, liver and kidney tissues.
**Fig. S7.** (A) Oncogenes and Tumor Suppressor Genes RT^2^ Profiler PCR Array.
**Fig. S8.** (A) Cancer Drug Target RT^2^ Profiler PCR Array.

## Data Availability

The datasets used and/or analyzed during the current study are available from the corresponding author on reasonable request.
