# Supplementary material for: TRAIL‐PEG‐Apt‐PLGA nanosystem as an aptamer‐targeted drug delivery system potential for triple‐negative breast cancer therapy using in vivo mouse model
Source: Mol Oncol. 2026 Feb 8;20(7):1742–61. doi: 10.1002/1878-0261.70202 (PMC13352959; doi:10.1002/1878-0261.70202)
Supplement: Supplementary file 1 — Table S1. PLGA nanoparticles (without TRAIL). Table S2. PLGA nanoparticles (with TRAIL). Table S3. Particle size, polydispersity index and zeta potential measurement results of PLGA nanoparticles (without TRAIL). Table S4. Characterization results of ideal PLGA nanoparticles (without TRAIL). Table S5. Characterization results of ideal PLGA nanoparticles (with TRAIL). [file MOL2-20-1742-s002.docx]

**Supplemantary Tables**

**Table 1.** PLGA nanoparticles (without TRAIL)

| Sol | Con | F1 | F2 | F3 | F4 | F5 | F6 | F7 | F8 | F9 |
| --- | --- | --- | --- | --- | --- | --- | --- | --- | --- | --- |
| I | PLGA (mg) | 25 | 10 | 5 | 25 | 10 | 5 | 25 | 10 | 5 |
|  | Acetone (mL) | 1 | 1 | 1 | 1 | 1 | 1 | 1 | 1 | 1 |
| II | PVA (mg) | 25 | 25 | 25 | 10 | 10 | 10 | 5 | 5 | 5 |
|  | D. water (mL) | 2 | 2 | 2 | 2 | 2 | 2 | 2 | 2 | 2 |
| Preparation process steps | | Solution I on II; drop by drop with 30G syringe under 500 rpm stirring | | | | | | | | |
|  |  | %30 amp. 1 minute probe sonication | | | | | | | | |
|  |  | Vacuuming of acetonone with 500 rpm stirring | | | | | | | | |
|  |  | %70 amp. 1 minute probe sonication | | | | | | | | |

(Sol: Solution, Con:Content, D. Water: deionized water)

**Table 1 continue.** PLGA nanoparticles (without TRAIL)

| Sol | Con | F10 | | F11 | | F12 | F13 | | F14 | | | F15 | F16 | |
| --- | --- | --- | --- | --- | --- | --- | --- | --- | --- | --- | --- | --- | --- | --- |
| I | PLGA (mg) | 25 | | 25 | | 5 | 25 | | 25 | | | 25 | 25 | |
|  | Acetone (mL) | 1 | | 1 | | 1 | 1 | | 1 | | | 1 | 1 | |
| II | PVA (mg) | 200 | | 100 | | 200 | 500 | | 500 | | | 100 | 100 | |
|  | D. water (mL) | 2 | | 2 | | 2 | 2 | | 2 | | | 2 | 2 | |
| Preparation process steps | | Solution I on II; drop by drop with 30G syringe under 500 rpm stirring | | | | | | | | | | | | |
|  |  | %30 amp. 1 minute probe sonication | | | | | | | | | | | | |
|  |  | Vacuuming of acetonone with 500 rpm stirring | | | | | | | | | | | | |
|  |  |  |  |  |  |  |  |  |  |  |  |  |  |  |
| Probe sonication | | %70,  1 min | %70,  1 min | | %70,  1 min | | | %70,  1 min | | %40,  1 min | %40,  1 min | | | %70, 30 sec |

(Sol: Solution, Con:Content, D. Water: deionized water)

**Table 2.** PLGA nanoparticles (with TRAIL)

| Sol | Con | 1T | 4T | 11T | 1TY | 4TY | 11TY | 11Tb4Y |
| --- | --- | --- | --- | --- | --- | --- | --- | --- |
| I | PLGA (mg) | 25 | 25 | 25 | 25 | 25 | 25 | 25 |
|  | Acetone (mL) | 1 | 1 | 1 | 1 | 1 | 1 | 1 |
| II | PVA (mg) | 25 | 10 | 100 | 25 | 10 | 100 | 100 |
|  | D. water (mL) | 2 | 2 | 2 | 2 | 2 | 2 | 2 |
|  | TRAIL (ng) | 400 | 400 | 400 | 400 | 400 | 400 | 800 |
| Preparation process steps | | Solution I on II; drop by drop with 30G syringe under 500 rpm stirring | | | | | | |
|  |  | %30 amp. 1 minute probe sonication | | | | | | |
|  |  | Vacuuming of acetonone with 500 rpm stirring | | | | | | |
|  |  | %70 amp. 1 minute probe sonication | | | | | | |

(Sol: Solution, Con:Content, D. Water: deionized water)

**Table 3.** Particle size, polydispersity index and zeta potential measurement results of PLGA nanoparticles (without TRAIL)

| **Formulation** | **Baseline** | **Intercept** | **Particle size (nm)** | **PDI** | **Zeta Potential (mV)** |
| --- | --- | --- | --- | --- | --- |
| F1 | 1,000 | 0,9086 | 281,0±5,53 | 0,076±0,051 | -7,0±0,5 |
| F2 | 0,994 | 0,9011 | 295,9±4,49 | 0,057±0,045 | -7,2±0,3 |
| F3 | 0,998 | 0,8971 | 336,1±17,02 | 0,161±0,056 | -13,4±1,5 |
| F4 | 0,997 | 0,9233 | 278,8±5,12 | 0,092±0,052 | -7,7±0,3 |
| F5 | 0,997 | 0,9148 | 321,0±7,59 | 0,131±0,075 | -5,5±0,3 |
| F6 | 1,002 | 0,9103 | 304,3±8,83 | 0,108±0,061 | -4,7±0,3 |
| F7 | 0,992 | 0,9208 | 279,5±6,21 | 0,076±0,041 | -8,5±0,3 |
| F8 | 0,998 | 0,9036 | 307,4±7,38 | 0,169±0,044 | -8,6±0,4 |
| F9 | 0,998 | 0,9145 | 294,8±5,28 | 0,067±0,039 | -7,9±0,4 |
| F10 | 1,016 | 0,8841 | 337,1±10,95 | 0,149±0,049 | -12,8±0,7 |
| F11 | 0,996 | 0,9010 | 276,3±7,86 | 0,094±0,050 | -12,6±0,4 |
| F12 | 1,136 | 0,9261 | 398,2±12,98 | 0,225±0,032 | -13,1±0,6 |
| F13 | 1,229 | 0,9167 | 564,8±53,14 | 0,268±0,042 | -21,7±1,0 |
| F14 | 1,085 | 0,8685 | 509,9±109,95 | 0,219±0,040 | -20,8±1,1 |
| F15 | 1,031 | 0,8577 | 1159,7±131,43 | 0,269±0,029 | -19,2±0,4 |
| F16 | 1,019 | 0,9224 | 1151,6±141,0 | 0,297±0,039 | -20,2±0,2 |

F: Formulation, PDI: polydispersity index

**Table 4**. Characterization results of ideal PLGA nanoparticles (without TRAIL)

| **Formulation** | **PLGA (mg)** | **PVA (mg)** | **Particle size (nm)** | **PDI** | **Zeta Potential (mV)** |
| --- | --- | --- | --- | --- | --- |
| F1 | 25 | 25 | 281,0±5,53 | 0,076±0,051 | -7,0±0,5 |
| F4 | 25 | 10 | 278,8±5,12 | 0,092±0,052 | -7,7±0,3 |
| F11 | 25 | 100 | 276,3±7,86 | 0,094±0,050 | -12,6±0,4 |

F: Formulation, PDI: polydispersity index

**Table 5.** Characterization results of ideal PLGA nanoparticles (with TRAIL)

| **Formulation** | **PLGA (mg)** | **PVA (mg)** | **Particle size (nm)** | **PDI** | **Zeta Potential (mV)** |
| --- | --- | --- | --- | --- | --- |
| 1T | 0,8870 | 1,011 | 343,0±13,36 | 0,211±0,075 | -1,3±0,7 |
| 4T | 0,9169 | 1,002 | 347,2±12,15 | 0,181±0,056 | -2,7±0,4 |
| 11T | 0,9210 | 1,031 | 410,2±24,67 | 0,202±0,077 | -3,0±0,6 |
| 11T-Apt | 0,9315 | 1,034 | 473,25±22,03 | 0,224±0,019 | -8,0±0,5 |

F: Formulation, PDI: polydispersity index
