# Supplementary material for: TRAIL‐PEG‐Apt‐PLGA nanosystem as an aptamer‐targeted drug delivery system potential for triple‐negative breast cancer therapy using in vivo mouse model
Source: Mol Oncol. 2026 Feb 8;20(7):1742–61. doi: 10.1002/1878-0261.70202 (PMC13352959; doi:10.1002/1878-0261.70202)
Supplement: Supplementary file 2 — Figure S1. The efficiency of protein TRAIL purification and fractionation. Fig. S2. Cell viability of TRAIL‐free nanosystems. Fig. S3. Cellular viability of MDA‐MB‐231 cells to TRAIL‐based formulations and paclitaxel for 12 h and 48 h. Fig. S4. Microscope images of CV staining. Fig. S5. Total weight changes and tumor weights in in NOD/SCID gamma mice. Fig. S6. HE staining images of lung, liver and kidney tissues. Fig. S7. (A) Oncogenes and Tumor Suppressor Genes RT2 Profiler PCR Array. Fig. S8. (A) Cancer Drug Target RT2 Profiler PCR Array. [file MOL2-20-1742-s001.docx]

**Supplementary Figures**


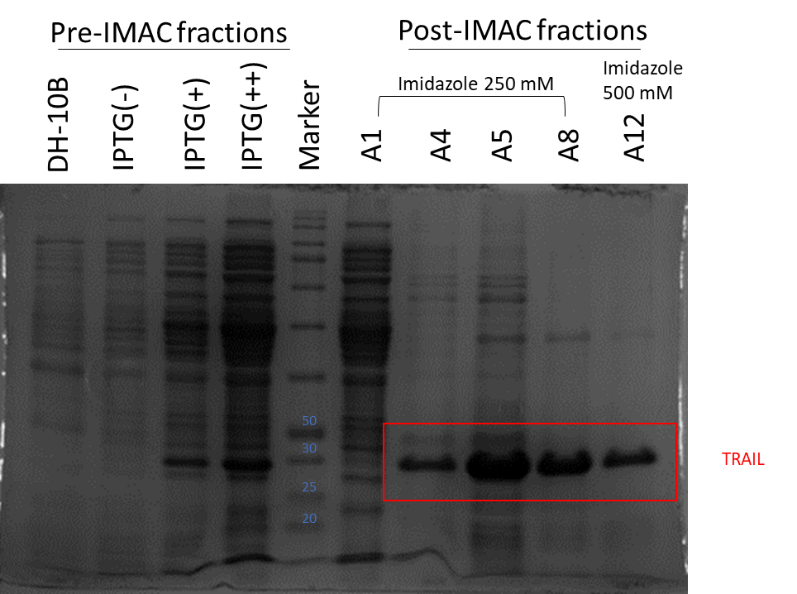


**Figure S1.** The efficiency of protein TRAIL purification and fractionation. Purification of TRAIL protein using Ni–NTA IMAC on a 16 mm × 25 mm column (Bio-Rad) with an NGC™ 10 chromatography system. The protein separation efficiency with imidazole concentration (250-500 mM) in 17% SDS-PAGE.


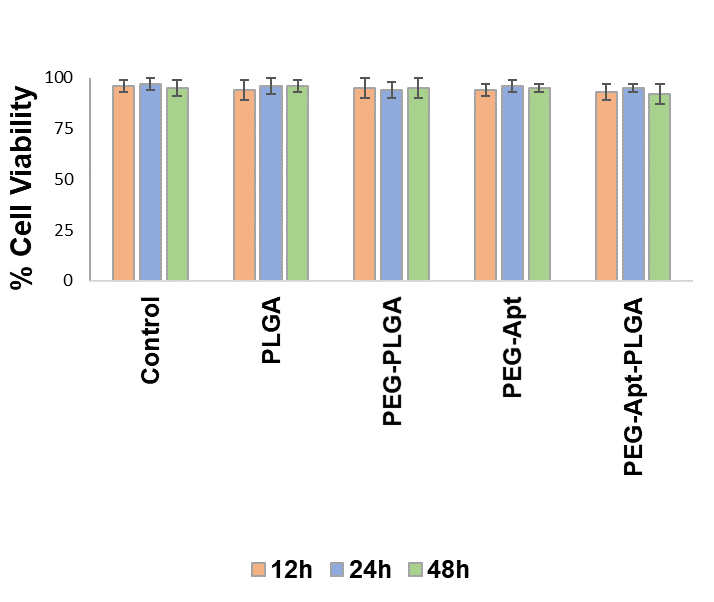


**Figure S2.** Cell viability of TRAIL-free nanosystems. Cell viability graphs of TRAIL-free nanosystems. Percent of cell viability of PLGA (10 µg/mL), PEG-PLGA (10 µg/mL), PEG-Apt (10 µg/mL), and PEG-Apt-PLGA (10 µg/mL) at 12, 24 and 48 hours of MDA-MB-231 cells. The data are presented as the mean±SD from three independent experiments.


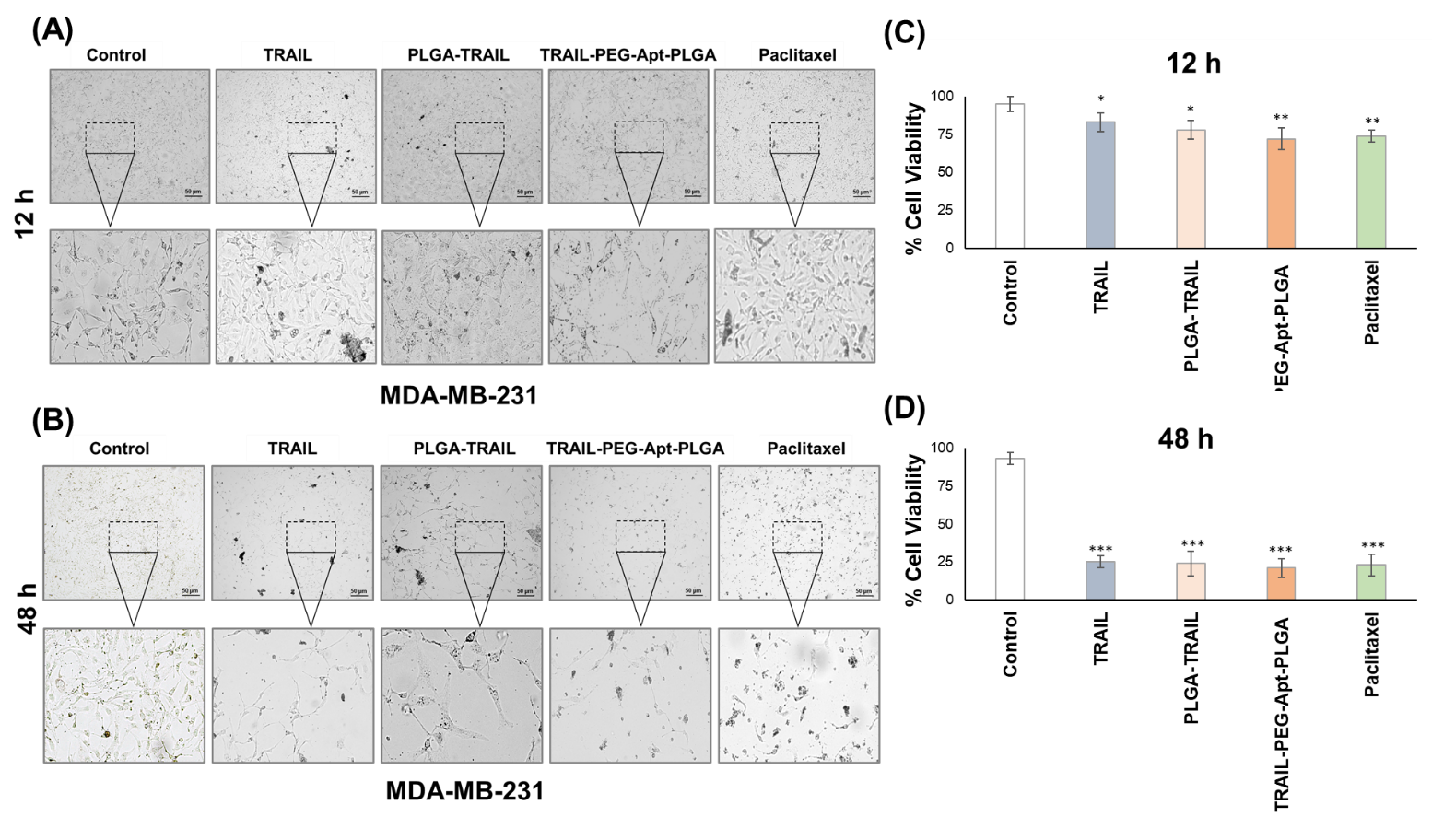


**Figure S3.** Cellular viability of MDA-MB-231 cells to TRAIL-based formulations and paclitaxel for 12 h and 48 h. Comparative treatments of MDA-MB-231 cells with TRAIL (10 ng/mL), PLGA-TRAIL (10 µg/mL), TRAIL-PEG-Apt-PLGA (10 µg/mL), and paclitaxel (10 nM). (A,C) Representative cell morphology images at 12 h and 48 h, respectively (Scale bar 50 µm). (B,D) Cell viability after 12 h and 48 h treatments, respectively. The data are presented as the mean±SD from three independent experiments. The statistical signiﬁcance were determined using Mann–Whitney U-test (***p˂0.001, **p˂0.01, *p˂0.05 compared to tumor group).


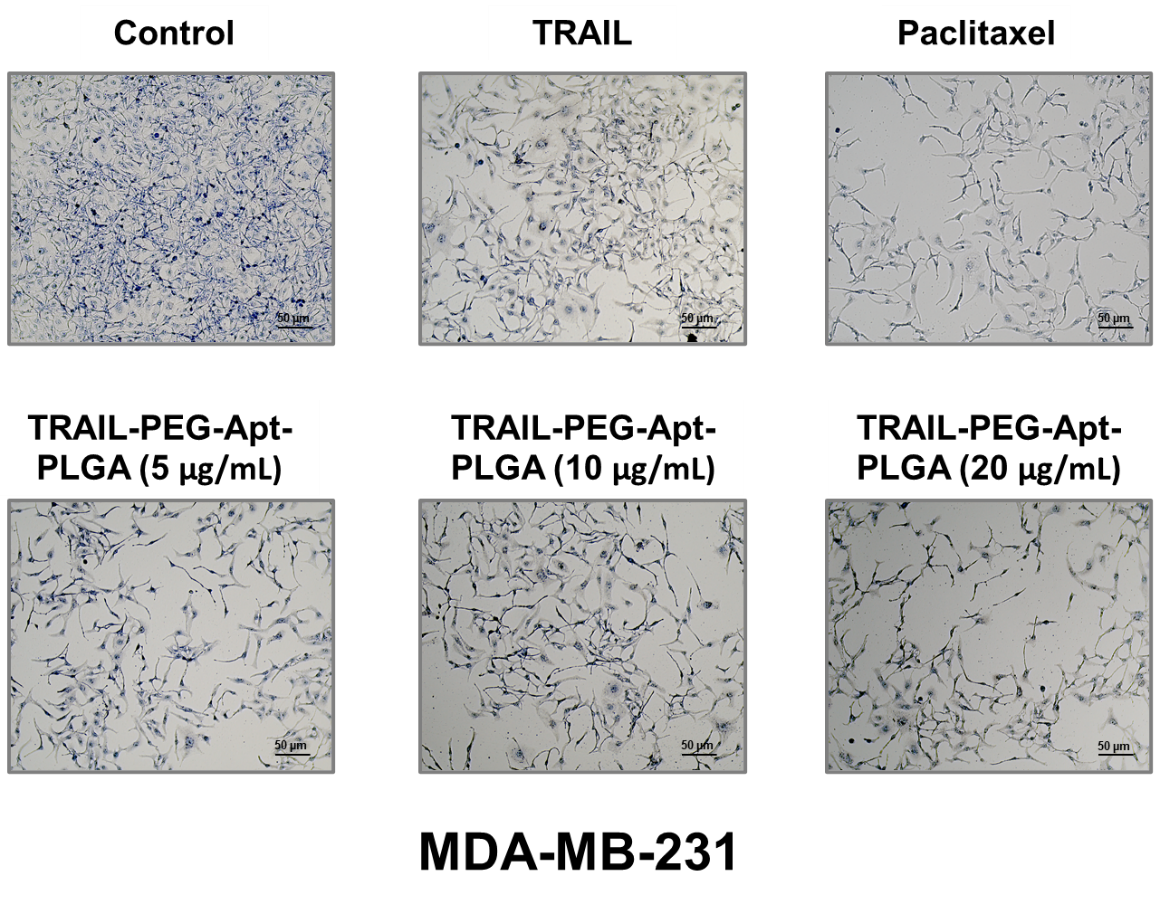


**Figure S4.** Microscope images of CV staining. Colony formation and cellular interaction of MDA-MB-231 cells treated with TRAIL (10 ng/mL), TRAIL-PEG-Apt-PLGA (5, 10, and 20 µg/mL), and paclitaxel (10 nM) for 14 days (Scale bar 50 µm).


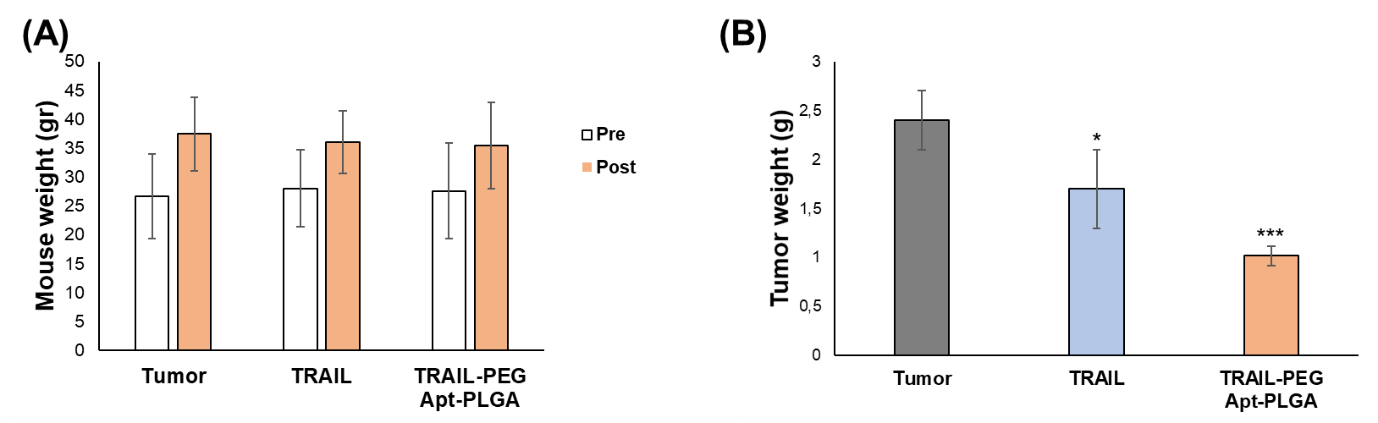


**Figure S5.** Total weight changes and tumor weights in NOD/SCID gamma mice. Changes in total body weight after treatment and (B) tumor weights of mice treated with free TRAIL (100 ng/kg) or TRAIL–PEG–Apt–PLGA (100 µg/kg). The data are presented as the mean±SD from three independent experiments. The statistical signiﬁcance were determined using Mann–Whitney U-test (***p˂0.001, *p˂0.05 compared to tumor group).


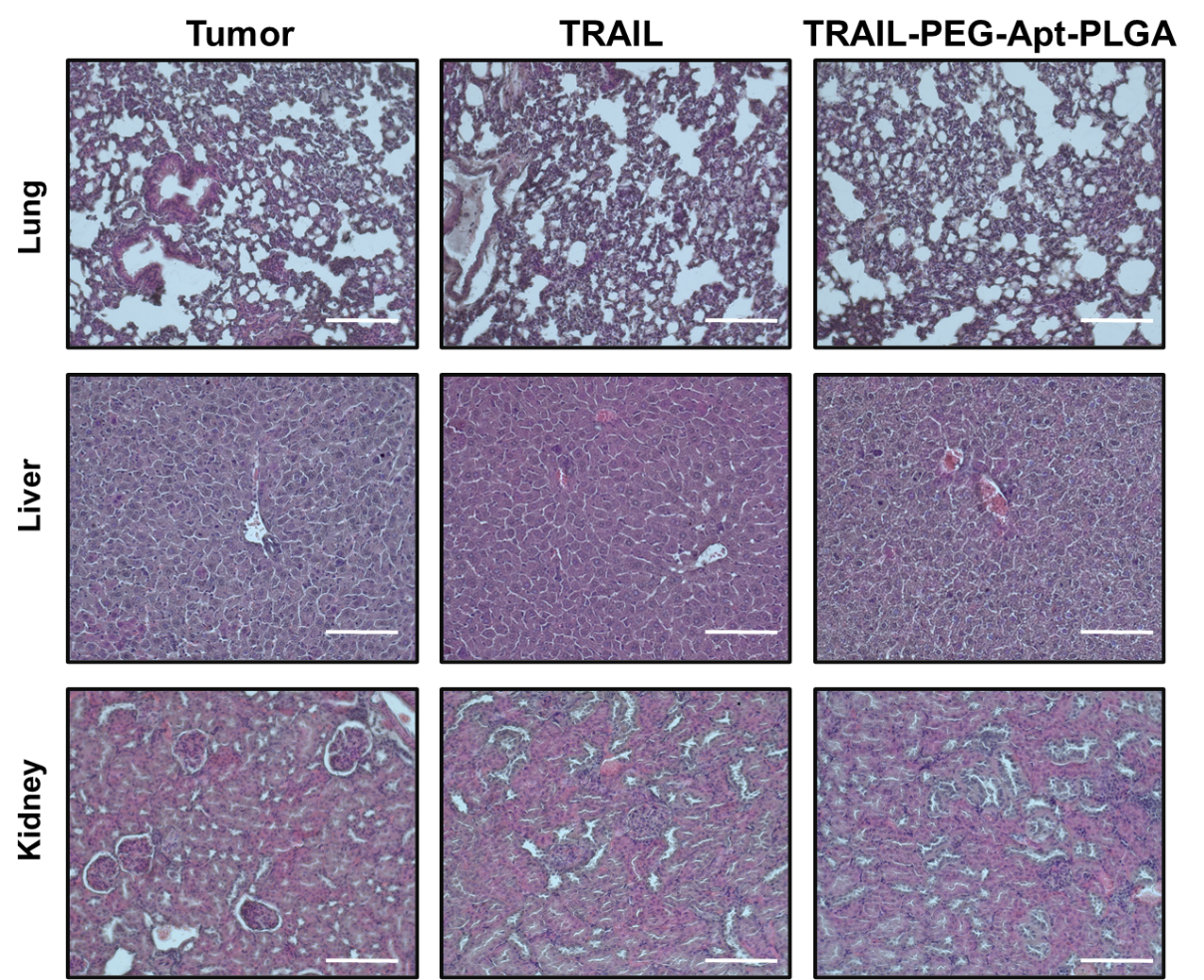


**Figure S6.** HE staining images of lung, liver and kidney tissues. HE staining of lung (top) (liver middle) and kidney (bottom) tissues colleceted 25 days after intravenous injection of TRAIL (100 ng/kg) or TRAIL-PEG-Apt-PLGA (100 µg/kg) in NOD/SCID gamma mice (Scale bar 50 µm).


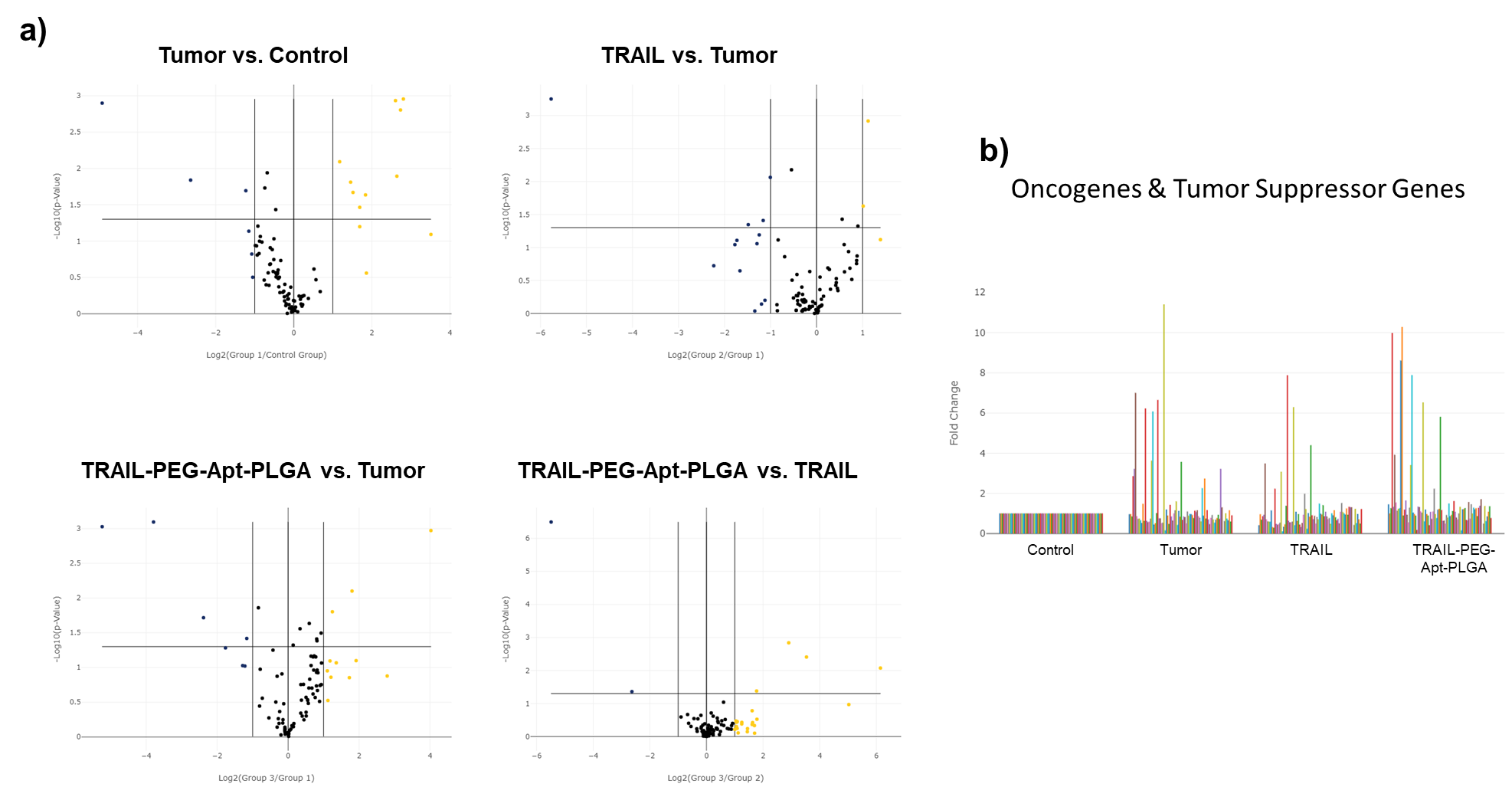


**Figure S7**. Oncogenes and Tumor Suppressor Genes RT^2^ Profiler PCR Array (A) Volcano plot analyses and (B) genes showing changes between groups.


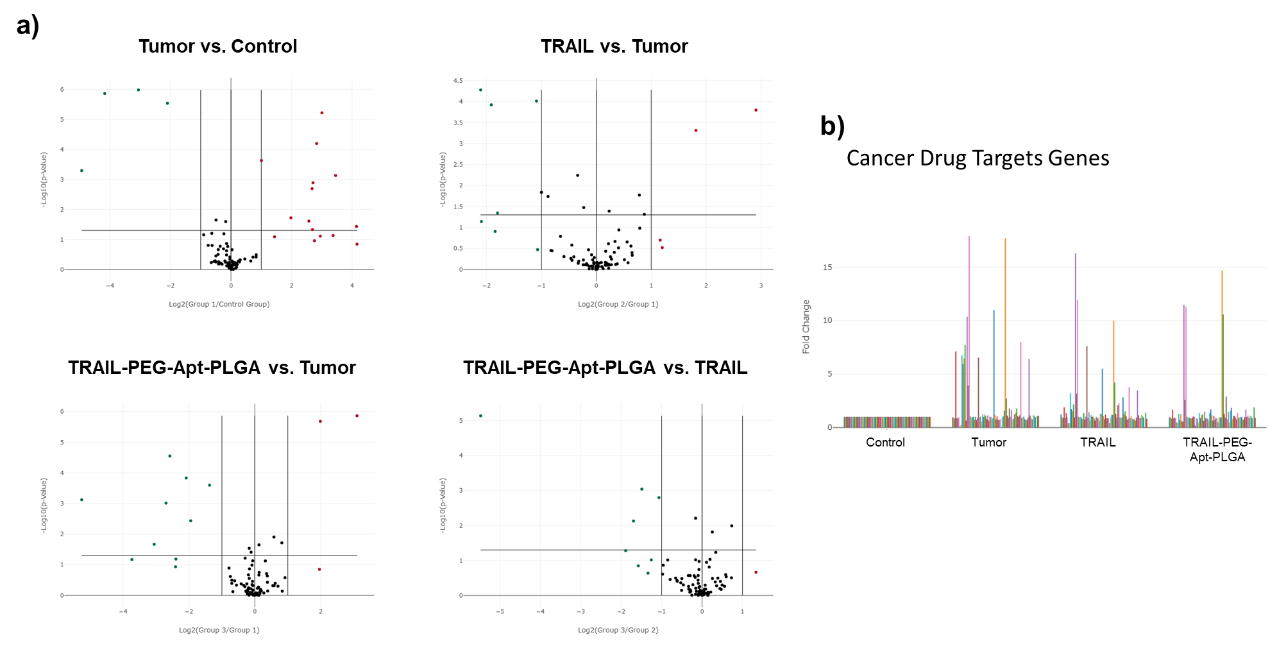


**Figure S8.** Cancer Drug Target RT^2^ Profiler PCR Array (A) Volcano plot analyses and (B) genes showing changes between groups.
